# From Synthesis to Characterization of Site-Selective PEGylated Proteins

**DOI:** 10.3389/fphar.2019.01450

**Published:** 2019-12-18

**Authors:** Lisandra Herrera Belén, Carlota de Oliveira Rangel-Yagui, Jorge F. Beltrán Lissabet, Brian Effer, Manuel Lee-Estevez, Adalberto Pessoa, Rodrigo L. Castillo, Jorge G. Farías

**Affiliations:** ^1^Department of Chemical Engineering, Faculty of Engineering and Science, Universidad de La Frontera, Temuco, Chile; ^2^Department of Biochemical and Pharmaceutical Technology, School of Pharmaceutical Sciences, University of Sao Paulo, Sao Paulo, Brazil; ^3^Faculty of Health Sciences, Universidad Autónoma de Chile, Temuco, Chile; ^4^Department of Internal Medicine East, Faculty of Medicine, University of Chile, Santiago de Chile, Chile

**Keywords:** protein PEGylation, site-selective conjugation, N-terminal PEGylation, enzymatic ligation, characterization

## Abstract

Covalent attachment of therapeutic proteins to polyethylene glycol (PEG) is widely used for the improvement of its pharmacokinetic and pharmacological properties, as well as the reduction in reactogenicity and related side effects. This technique named PEGylation has been successfully employed in several approved drugs to treat various diseases, even cancer. Some methods have been developed to obtain PEGylated proteins, both in multiple protein sites or in a selected amino acid residue. This review focuses mainly on traditional and novel examples of chemical and enzymatic methods for site-selective PEGylation, emphasizing in N-terminal PEGylation, that make it possible to obtain products with a high degree of homogeneity and preserve bioactivity. In addition, the main assay methods that can be applied for the characterization of PEGylated molecules in complex biological samples are also summarized in this paper.

## Introduction

**Figure 1:**
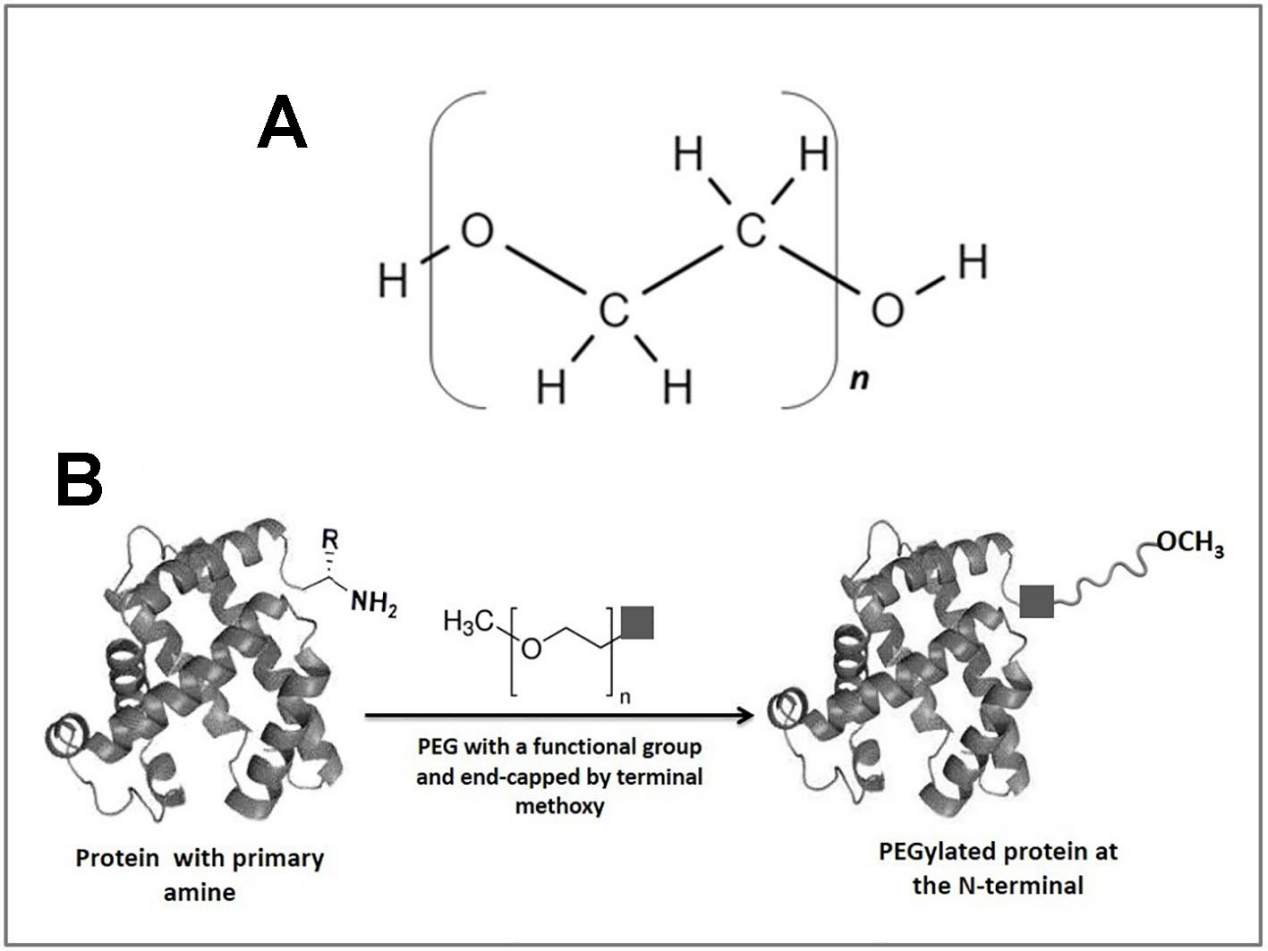
A general scheme of protein PEGylation at N-terminus. **(A)** PEG chemical structure based on linear polyethylene oxide repeating units. **(B)** Schematic representation of the N-terminal PEGylation reaction. The square represents PEG functional group that covalently binds to the terminal amine of the protein. PEG chain is end-capped with a terminal methoxy group to prevent reactivity and enzymatic attack upon administration in mammals.

The binding of proteins, peptides, enzymes, antibody fragments, oligonucleotides, or small synthetic drugs to polymers has become a very useful method for improving therapeutic activity or decreasing the toxicity of these biological agents (Mishra et al., 2016). Among the polymeric materials, polyethylene glycol (PEG) is the most used for these purposes, mainly due to its high biocompatibility, low toxicity, and limited side effects (Gauthier and Klok, 2008). PEGs are water-soluble polymers approved by the Food and Drug Administration for use in oral, topical, and intravenous formulations (D’souza and Shegokar, 2016). It presents a structure of repeated units of polyether diols (either linear or branched) chemically formulated as HO-(CH_2_CH_2_O)n-CH_2_CH_2_-OH (**Figure 1A**), where each ethylene oxide residue has a molecular weight (MW) of 44 Da (Roberts et al., 2012). PEGylation refers to the covalent or non-covalent attachment of PEG to different molecules, such as proteins, macromolecular carriers, oligonucleotides, vesicles, and others to improve the pharmacokinetic (Milton Harris et al., 2001; Lee et al., 2013) and pharmacodynamic properties (Abbina and Parambath, 2018). The conjugation to PEG generates an increase in the hydrodynamic volume of the biomolecule of interest, creating a shield around it (Gokarn et al., 2012). This effect enables clearance by the renal system to be reduced, and therefore, the half-life is increased in the bloodstream (Milton Harris and Chess, 2003) concomitant with the increases in PEG molecular weight (Hamidi et al., 2006). Additionally, this approach has been used to improve the stability of some proteins (Yang et al., 2007; Jevševar et al., 2010; Lawrence and Price, 2016; Santos et al., 2019), as well as decrease the immune response against several biomolecules (Soares et al., 2002; Zheng et al., 2012; Sathyamoorthy and Magharla, 2017; Wu et al., 2017).

Since the 1990s several PEGylated biopharmaceuticals (see **Table 1**) have been approved by the FDA, and some more are currently undergoing clinical trials (information available at https://clinicaltrials.gov/ct2/results?term=Pegylated&Search=Apply&recrs=d&age_v=&gndr=&type=&rslt=). Most of the approved PEGylated proteins were synthesized by non-site-specific chemical conjugation strategies, resulting in heterogeneous mixtures of multi-PEGylated (polydisperse) proteins due to the presence of several reactivity sites on the protein surface (Alconcel et al., 2011), requiring complex separation steps. In addition, protein PEGylation can lead to the loss of protein activity through several mechanisms that include the direct PEGylation of the active site or receptor binding site (Schiavon et al., 2000), the steric entanglement imposed by PEG chains that cause restricted movements (Kubetzko, 2005; Xiaojiao et al., 2016) and conformational changes in proteins (Chiu et al., 2010), among others. Also, recent research has revealed certain shortcomings related to highly PEGylated forms, such as activation of the immune system, non-degradability, and possible accumulations with high molecular weight PEGs. These are strong reasons that support the need to find site-selective PEGylation techniques, yielding homogenous mono-PEGylated products, a field that has garnered considerable in recent years. Although certainly the *in vivo* potency of therapeutic proteins can be affected by the PEGylation process, this decrease in activity can be largely balanced by their prolonged half-life in the circulation (Oclon et al., 2018). Site-selective PEGylation has been a very useful strategy for introducing PEG at specific amino acid sites in various proteins. Some methods like pH-controlled N-terminal selective acylation (Chan et al., 2006; Chan et al., 2012) or reductive alkylation (Kinstler et al., 1996; Marsac et al., 2006), the use of oxidizing agents (Kung et al., 2013; Obermeyer et al., 2014), the chemo-selective capability of catechol (Song et al., 2016) and transamination reaction (Gilmore et al., 2006) have been used to perform PEGylation at the N-terminus of proteins. Additionally, in recent years there has been a lot of work on using “grafting from” approaches to grow PEG from the surface of proteins *via* ATRP and RAFT polymerization methods (Quémener et al., 2006; Ameringer et al., 2013; Gody et al., 2015; Tucker et al., 2017). These approaches involve the direct generation of conjugates containing high molecular weight polymers (like PEGs) by directly growing the polymer from the protein surface (Wallat et al., 2014; Obermeyer and Olsen, 2015).

**Table 1:** PEGylated therapeutic peptides and proteins approved for clinical applications.

Product names	PEGylated drugs	Sites of Attachment	PEG sizes	Indications	Manufacturers	References
PEG-ADA (Adagen^®^)	PEGylated bovine adenosine deaminase	Lysines, N-terminal	5 kDa	Imunodeficiency disease caused by adenosine deaminase deficiency	Enzon Pharmaceuticals Inc., USA	(Lainka et al., 2005; Alconcel et al., 2011)
PEGASPARGASE (Oncaspar^®^)	PEGylated L-asparaginase	Lysines, serine, tyrosine,hystidine	5 kDa	Acute lymphoblastic leukemia	Enzon Pharmaceuticals Inc., USA	(Ettinger, 1995)
PEGVISOMANT (Somavert^®^)	PEGylated recombinant receptor antagonist of the human growth hormone	Lysines 38,120,140,158, N-terminal phenylalanine	5 kDa (4–6 units)	Acromegaly	Pharmacia & Upjohn Company LLC., USA	(Kochendoerfer, 2003; Alconcel et al., 2011)
PEG-INTRON^®^	PEGylated interferon alfa-2b	Hystidine 34 (major)	12 kDa	Chronic hepatitis C	Schering-Plough Corporation, USA	(Wang et al., 2002; Alconcel et al., 2011)
PEGASYS^®^	PEGylated interferon alfa-2a	Lysines 31, 121, 131, or 134	40 kDa	Chronic hepatitis C & B	Hoffmamn-La Roche Inc., USA	(Foser et al., 2003)
CERTOLIZUMAB PEGOL (Cimzia^®^)	PEGylated tumor necrosis factor alpha inhibitor	C-Terminal cysteine	40 kDa	Crohn’s disease	UCBPharma, Belgium	(Gunnoo and Madder, 2016)
PEGFILGRASTIM (Neulasta^®^)	PEGylated granulocyte colony-stimulating factor analog	N-Terminal methionine	20 kDa	Neutropenia during chemotherapy	Amgen Inc., USA	(Molineux, 2004)
PLEGRIDY^®^	PEGylated β-interferon 1a	N-terminal	20 kDa	Multiple sclerosis	Biogen Idec, USA	(Turecek et al., 2016)
PEGLOTICASE (Krystexxa^®^)	PEGylated urate oxidase	Lysines	10 kDa (10–11 units)	Chronic gout	Horizon Pharmaplc, Dublin	(Alconcel et al., 2011; Lyseng-Williamson, 2011)
Mircera^®^	PEGylated erythropoietin receptor activator	Lysine 52 or 46	30 kDa	Anemia associated with chronic kidney disease	Roche Inc., USA	(Cano et al., 2011)
Sylatron^®^	PEGylated interferon alfa-2b	C-terminal cysteine	12 kDa	Adjuvant therapy in resected stage III melanoma	Merck Sharp & Dohme Corp, USA	(Mishra et al., 2016)
PEGINESATIDE** (Omontys^®^)	PEGylated synthetic peptide, erythropoietin analogs	Lysines	40 kDa	Anemia associated with chronic kidney disease	Affymax Inc, USA & Takeda Pharmaceutical Co, Japan	(Stidl et al., 2016)

**Recalled from the market on 2013 due to hypersensitivity.

An illustrative example of success in chemical site-selective PEGylation is the case of Neulasta^®^, which is an N-terminally mono-PEGylated granulocyte colony-stimulating factor bearing a 20-kDa PEG (Molineux, 2004). The improved pharmacokinetic behavior of this biopharmaceutical allows administration only once per chemotherapy cycle compared to the first generation, Neupogen^®^, which is administered daily (Cesaro et al., 2013; Zhang et al., 2015).

Despite their robustness, chemical methods usually involve the use of excessive amounts of reagents and careful working conditions. Site-specific PEGylation of peptides and proteins has been approached successfully not only from the chemical point of view but also enzymatically. Several studies report the use of enzymes to conjugate PEG to peptides, proteins, and oligonucleotides (Sato, 2002; Mero et al., 2009; Da Silva Freitas et al., 2013; Sosic et al., 2014).These enzymes usually catalyze the reaction between the biomolecule of interest and a substrate analog containing a functional group (Dozier and Distefano, 2015), which can be the case of PEG. There are a number of more recent approaches aimed at achieving site-selective modification including the use of the Spytag/Spycatcher system (Schoene et al., 2014; Reddington and Howarth, 2015; Cayetano-Cruz et al., 2018; Kim et al., 2018); however, there is as yet insufficient information focused on these methods, and some studies are being developed in this direction.

The structural changes in protein characteristics after the attachment to PEG influence the subsequent characterization of PEGylated proteins. These changes result in an analytical challenge due to the heterogeneity of the PEGylation products and the degree of PEGylation, coupled with the complex protein structure (Hutanu, 2014). Several studies have reported the use of analytical techniques with differing degrees of difficulty—from colorimetric methods to more complex techniques such as computational approaches—for the characterization of PEGylated peptides and proteins.

In the present review, we have focused on summarizing both classic and novel chemical and enzymatic tools used for the covalent attachment of PEG in site-specific regions of peptides and proteins, as well on the main analytical methods for PEGylated molecule characterization.

### Chemical Approaches for Site-Selective Pegylation

For the selective modification of specific amino acids in peptides and proteins, the knowledge of some characteristics about their primary structure is needed. An important physicochemical feature in proteins is the difference in pKa between the amino group of an N-terminal amino acid residue (∼7.6) and the amino groups in the side chains of lysine (∼10.5) and arginine (∼12) (Roberts et al., 2012). This difference allows the selective N-terminal modification of proteins based on pH control and the use of reductive agents like sodium cyanoborohydride. A useful strategy for the specific conjugation of peptides and proteins is based on the amino acid ratio in a protein being variable. Moelbert et al. reported the accessibility index on the surface of the 20 essential amino acids, which makes it possible to know the expression of these amino acids in different areas of the proteins in relation to their natural abundance (Moelbert, 2004). It has also been reported that short peptides/proteins (less than 50 residues) tend to over-represent glutamine and cysteine in the N-terminal region (Villar and Koehler, 2000). It is well known that single-chain proteins possess only one N-terminal residue, having a uniquely reactive site for chemical modification (Rosen and Francis, 2017). Therefore, as virtually all proteins present these functional groups, a number of valuable reactions have been developed for their selective modification (Boutureira and Bernardes, 2015).

The use of potassium ferricyanide as an oxidizing agent in o-aminophenol-performing N-terminal PEGylation has also been shown (Obermeyer et al., 2014). In 2016 Song et al. described an alternative strategy for PEGylation at the N-terminus of several proteins as well as two peptides based on the chemoselectivity of catechol (Song et al., 2016). More recently, Rosen and Francis described classical methods for the selective modification of N-terminal amino group under pH control. These methods include the selective acylation and alkylation of N-terminal amines at low-to-neutral pH and also transamination using pyridoxal-5′-phosphate aldehyde, which undergoes condensation with ε-amines from lysine side chains and N-terminal α amines to form imines (Gilmore et al., 2006; Rosen and Francis, 2017). Chen et al. demonstrated the ability of benzaldehyde to selectively modify native peptides and proteins on their N-termini. Preservation of the positive charge on the N-terminus of the human insulin A-chain through reductive alkylation instead of acylation leads to a 5-fold increase in bioactivity. They showed that under mild conditions, aldehyde derivatives and carbohydrates can site-specifically react with peptide and protein N-termini, providing a universal strategy for site-selective N-terminal functionalization in native peptides and proteins (Chen et al., 2017).

PEG-isocyanate is in the group of PEG reagents used for the site-specific modification of different proteins (Berberich et al., 2005; Sharma et al., 2017). The reaction takes place *via* the amine group to produce a stable thiourea linkage (Ganesan et al., 2015). For example, in 2009 Cabrales et al. generated PEGylated human serum albumin (PEG-HSA) by conjugating PEG-phenyl-isothiocyanate 3 and 5 kDa at primary amine groups of the HSA, enhancing the hydrodynamic volume of the protein and restoring intravascular volume after hemorrhagic shock resuscitation (Cabrales et al., 2008). Furthermore, Chen and He reported in 2015 the achievement of nanophosphors coated with PEG-isocyanate and polylactic acid (PLA) for paclitaxel delivery, resulting in a significant improvement and serving as a platform in the field of drug development (Chen and He, 2015). Lee et al. synthesized a dual functional cyclic peptide gatekeeper attached on the surface of nanocontainers by using PEG-isocyanate as a linker to enhance dispersion stability and biocompatibility. This allowed the active targeting of cancer cells with high CD44 expression together with the ability of triggered drug release (Lee et al., 2018). It is important to note that specific PEG-reagents like isocyanates have a short half-life in aqueous solutions (Erfani-Jabarian et al., 2012); thus, a stoichiometric excess of these reagents is necessary, causing difficulties in the removal of the remaining PEG.

A relevant report for one-step N-terminus-specific protein modification showed the stable and selective imidazolidinone product at the N-terminus, with 2-pyridinecarboxaldehyde (2PCA) derivatives (Macdonald et al., 2015). The main basis of this reaction is the nucleophilic attack of the neighboring amide nitrogen on the electrophilic carbon of the initially formed N-terminal imine (Koniev and Wagner, 2015). As an example, a 2PCA-functionalized polyacrylamide-based hydrogel has been developed for the immobilization of extracellular matrix proteins through the N-terminus to study their biochemical and mechanical influence on cells (Lee et al., 2016).

**Figure 2:**
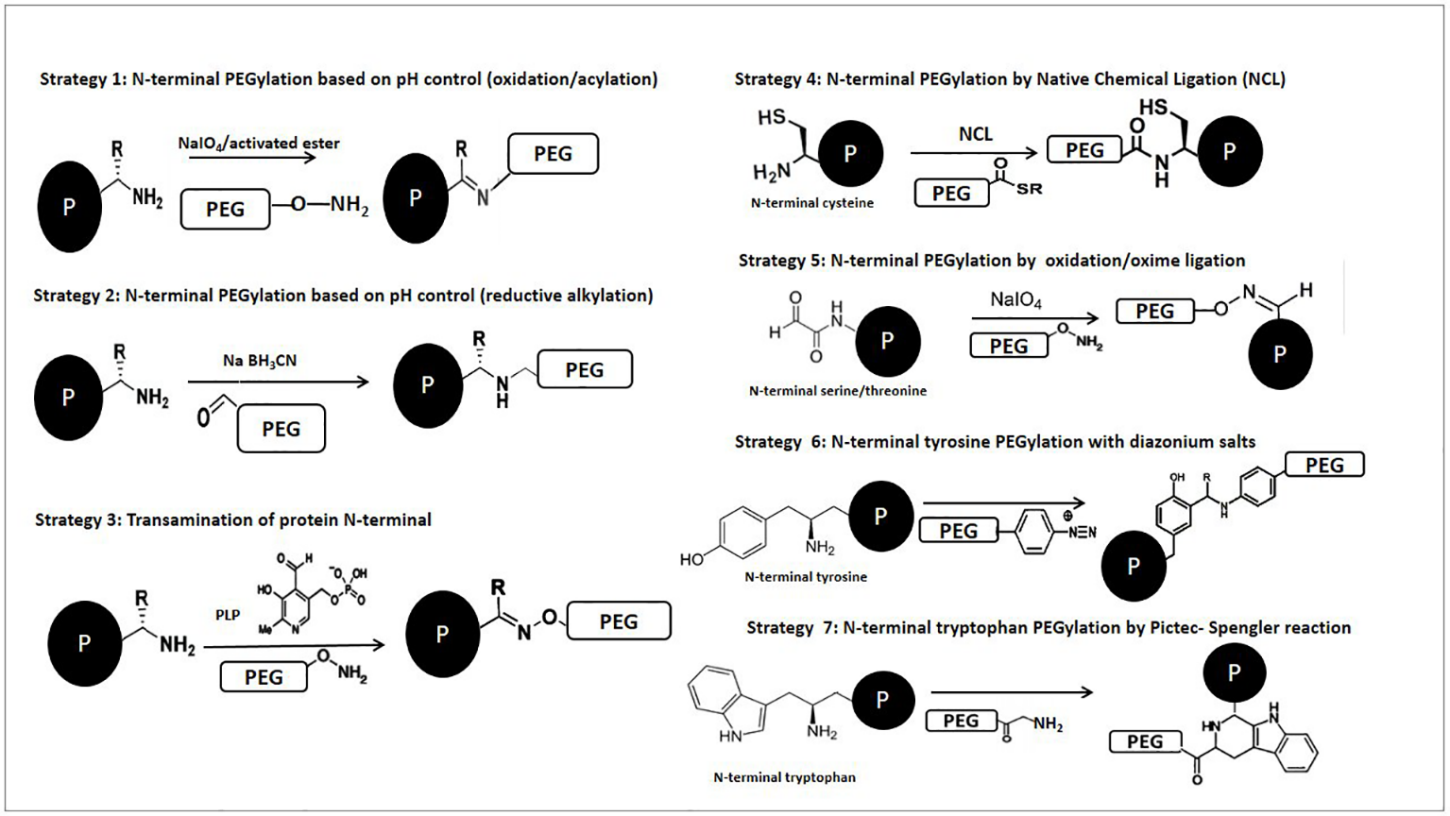
Schematic representation of chemical reactions described for the selective PEGylation of proteins.

In the next section, we provide an overview based on reactions which can be used to selectively modify specific amino acids. Keeping that in mind, in some cases the described modification does not refer to the PEGylation itself, but the concept could be applied if the introduction of PEG reagents is desired. A mechanism corresponding to N-terminal PEGylation has been illustrated in **Figure 1B**, while general mechanisms of the site-selective chemical reactions are shown in **Figure 2**.

### Strategies for the Modification of Specific Amino Acids

#### Targeting Cysteine

Cysteine residues are interesting targets for residue-specific modification of peptides/proteins due to their low apparition frequency (Harvey et al., 2000). These are often found partially or fully covered within the protein structure, limiting their accessibility to chemical reagents (Thordarson et al., 2006). Proteins with N-terminal cysteine have been successfully modified through native chemical ligation (NCL) when, on the first and reversible step, a thioester intermediate is formed, which then undergoes a spontaneous S-to-N acyl shift and yields an amide bond (Johnson and Kent, 2006; Rosen and Francis, 2017). This methodology has been useful in the preparation of high complexity protein–polymer conjugates. For example, Zhao et al. described a PEGylated human serum albumin (HSA) in a site-specific method by taking advantage of the unusual chemical reactivity of the only one free Cys34 of the HSA molecule and the high specificity of PEG-maleimide for the protein sulfhydryl (– SH) groups. Targeting the distinctive free Cys34 through this site-specific PEGylation could generate a chemically well-defined and molecularly homogeneous product and may be also convenient in preventing dimerization (Zhao et al., 2012). Another technique which plays a major role in modern chemical biology and has been used for many applications is known as expressed protein ligation (EPL) (Mitchell and Lorsch, 2014; David et al., 2015; Liu et al., 2017). EPL constitutes an improvement for NCL, and in this case selectivity over lysine acylation was achieved through pH control, by using benzaldehyde derivatives bearing selenoesters to acylate N-terminal positions through acyl transfer (Raj et al., 2015).

As N-terminal cysteines are rare in nature, they frequently need to be introduced by genetic engineering (Nguyen et al., 2014a; Uprety et al., 2014; Gunnoo and Madder, 2016). Methionine aminopeptidase can take out the first methionine to liberate an N-terminal cysteine (Gentle et al., 2004), and some proteolytic enzymes that specifically cleave in the presence of cysteine residues in a protease recognition sequence (Busch et al., 2008; Wissner et al., 2013) have been used as strategies for the exposure of N-terminal cysteine and its subsequent bioconjugation.

#### Targeting Serine and Threonine

The presence of an N-terminal serine or threonine offers unique opportunities due to the high susceptibility of 1, 2-aminoalcohols to periodate oxidation, resulting in the formation of a glyoxylyl group, which can be used to form several linkages (Xiao et al., 2005). It has been shown that the extra periodate used to oxidize the N-terminal residues of proteins carries the risk of oxidizing other residues, such as cysteines and methionines, as well as causing unwanted oxidative cleavage of protein glycosyl groups (Huang et al., 2015). This is mainly the approach applied in classical research, based on targeting serines or threonines at the N-terminal position, which uses periodate oxidation to generate a glyoxylyl group. Gaertner et al. performed site-selective PEGylation of an N-terminal serine residue, which was oxidized using sodium periodate followed by subsequent oxime ligation with an aminooxy and hydrazyde PEG derivative (Gaertner and Offord, 1996). The modified proteins, interleukin (IL)-8, granulocyte colony-stimulating factor (G-CSF) and IL-1rα, fully retained their activity after PEGylation (Krall et al., 2016).

#### Targeting Tyrosine

Francis et al. Have Reported a Number of Efficient strategies where tyrosine residues were modified *via* a three-component Mannich-type reaction, alkylation of the residue and coupling with diazonium reagents (Tilley and Francis, 2006). However, Jones et al. were the first to describe direct polymer conjugation, including PEGylation, to tyrosine residues. These authors developed a general route to polymer-peptide biohybrid materials by preferentially targeting peptide tyrosine residues using diazonium salt-terminated polymers. Also, aniline derivatives are attractive molecules for tyrosine-targeted protein modifications with 4-aminobenzoyl-N-PEG_2000_-OMe through either diazonium coupling or three-component Mannich-type reactions (Jones et al., 2012). Recently, the first study to apply Mannich reaction modification and reactive coloration in fibrous proteins was developed, providing promising future applications for the reactive dyeing process of silk (Chen et al., 2019).

#### Targeting Tryptophan

Peptides containing N-terminal tryptophan residues may be modified using the Pictet-Spengler reaction with aldehydes in glacial acetic acid. The Pictet-Spengler reaction is based on the oxidation of the N-terminal amino group to an imine, where an aldehyde undergoes cyclic condensation with the α-amine and the indole side chain of a tryptophan residue, forming a new stable C–C bond (Agrawal et al., 2013; Mittal et al., 2014). Li et al. applied the Pictet-Spengler reaction to peptide ligation using peptide segments containing an aldehyde at the C-terminal and a Trp at the N-terminal. The main advantage of this reaction is the formation of a product with a stable C–C bond in a single step (Li et al., 2000). Also, Sasaki et al. applied the Pictet-Spengler reaction to the N-terminal labeling of horse heart myoglobin with an N-terminal glycine, employing tryptophan methyl ester and tryptamine as the coupling partners (Sasaki et al., 2008).

As an alternative to chemoselective modification, recombinant methods have also been used to incorporate unnatural amino acids (UAA) into proteins as chemical handles for a bio-orthogonal conjugation reaction (Liu and Schultz, 2010). The transfer of non-natural amino acids with azide and ketone functional groups at the N-terminus of proteins bearing N-terminal arginine residues using leucyl/phenylalanyl (L/F)-tRNA-protein transferase has proven efficient, both in the presence of other peptides and in crude protein mixture (Taki and Sisido, 2007). Although considerable progress has been made, an improvement in the existing N-terminal strategies is needed as none of the methods reported to date offer universal sequence compatibility.

## Enzymatic Tools for Selective Pegylation of Proteins

**Figure 3:**
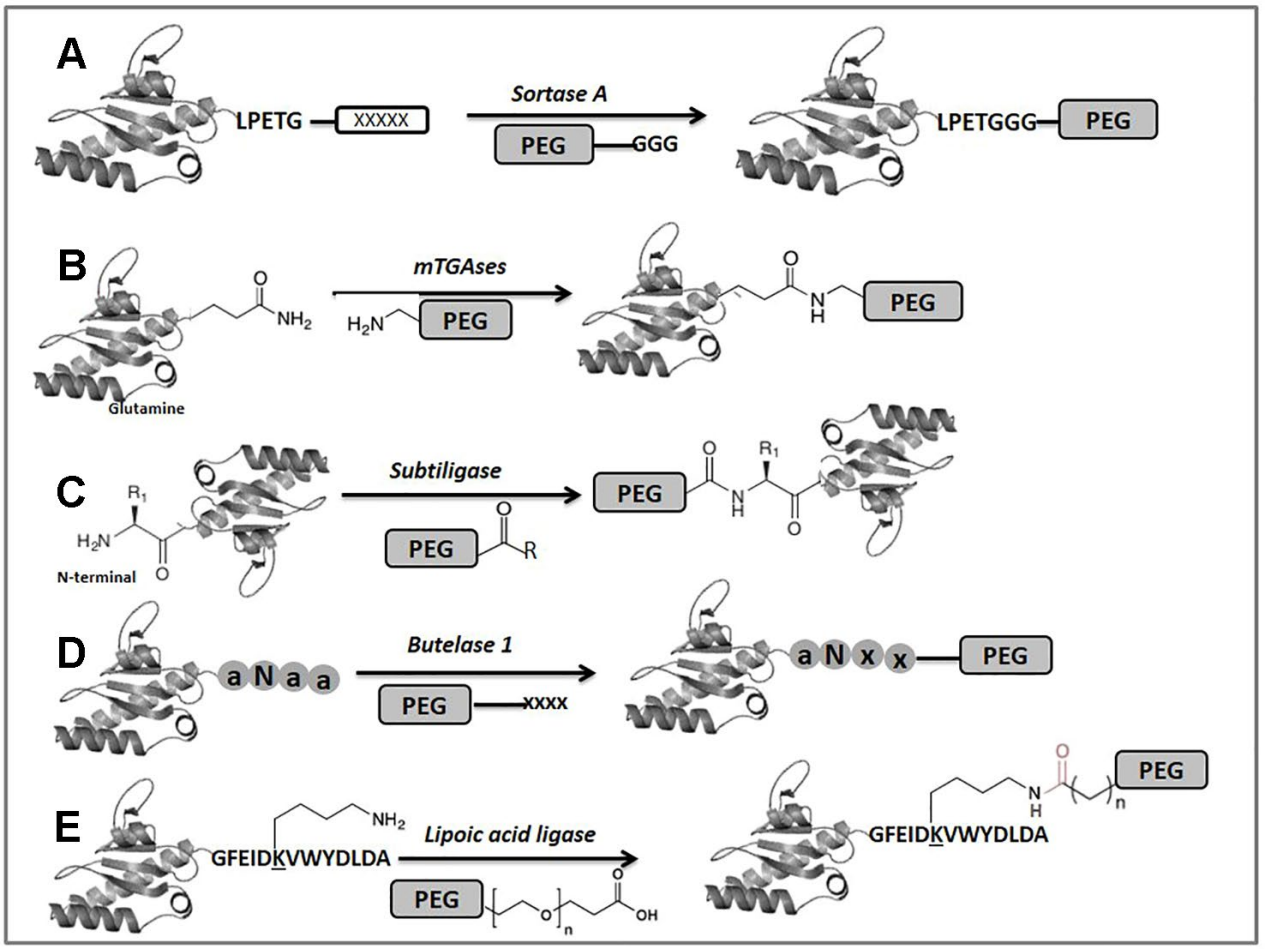
Enzyme-mediated modification of proteins. The (x) and (a) locked in circles represent hypothetical amino acids.

Enzyme-mediated bioconjugation has gained a lot of attention in recent years because of the ability of biocatalysts to modify specific molecular tags under mild conditions. In this section, we briefly explore some enzymatic tools used for selective PEGylation purposes. Among these, *sortase A* (SrtA) from *Staphylococcus aureus* has been the most widely applied enzyme for protein bioconjugation in academic research (Tsukiji and Nagamune, 2009; Popp and Ploegh, 2011; Schmidt et al., 2017; Wang et al., 2017). It catalyzes a transpeptidase reaction between an N-terminal amino group derived from glycine and a specific internal amino acid sequence on a protein, usually LPXTG (where X can be any amino acid) (Rosen and Francis, 2017) (**Figure 3A**). Although the *sortase A* is applied for labeling the peptides and proteins among them, the approach of *sortase*-mediated PEGylation has been used to label large macroscopic particles with PEG-stabilized proteins to the surface of cells (Tomita et al., 2013). More recently, Li et al. took advantage of the mutated *sortase A* enzyme, which can enzymatically ligate the universal α-amino acids to a C-terminal tagged protein, allowing specific modification of the C-terminus of human growth hormone (hGH) with PEG. This site-specific bound PEG-hGH has similar efficacy as wild-type hGH (Shi et al., 2018). Despite there being as yet no approved PEGylated drugs derived from sortagging, it could be a promising advancement for improving the performance of traditional PEGylated drugs.

### Microbial Transglutaminases

Microbial transglutaminases (mTGases) are another class of enzymes that has frequently been used for protein conjugation (**Figure 3B**). Several excellent reviews covering applications of microbial transglutaminase have been published previously (Mariniello and Porta, 2005; Rachel and Pelletier, 2013; Adrio and Demain, 2014; Strop, 2014). In general terms, TGases catalyze the acyl transfer reaction between the c-carboxyamide group of a protein-bound Gln residue and a variety of linear primary amines, such as the amino group of Lys (Griffin et al., 2002). In terms of site selective PEGylation this approach could be ineffective due to promiscuity in the amine substrates for these enzymes (Rachel and Pelletier, 2013). Nevertheless, Pasut et al. examined how the properties of PEGylated human growth hormone (hGH) changed depending on whether it was generated by chemical modification at the N-terminus or enzymatically using transglutaminase. Enzymatic labeling of hGH was carried out using TGase and a PEG reagent incorporating a primary amine. The study shows that although hGH carries 13 glutamine residues, 63.3% of the reaction product was a monoPEGylated form at position 141, showing a certain degree of site selectivity (Da Silva Freitas et al., 2013). Spolaore et al. studied the reactivity of IFN α-2b to microbial mTGase to obtain a site-specific conjugation of this biopharmaceutical. Characterization by mass spectrometry of the conjugates indicated that among the 10 Lys and 12 Gln residues of the protein only Gln101 and Lys164 were selectively conjugated with a PEG-NH_2_ for Gln101 and a PEG modified with carbobenzoxy--glutaminyl-glycine for Lys164 derivatization, with activity retention and improvements at pharmacokinetic levels (Spolaore et al., 2016). A mono-PEGylated derivative of filgrastim (granulocyte colony-stimulating factor) was also prepared using mTGase. The conjugation yielded an active protein with a single conjugation site (Gln135) that exhibited good *in vivo* stability (Scaramuzza et al., 2012). Although in the previous examples the PEGylation sites do not correspond to the N-terminal amino acid, they do illustrate a partial selectivity of mTGase despite its tendency toward substrate promiscuity. Also, these results indicate the potential of mTGase in the future of specific PEGylation and the development of innovative biopharmaceuticals. More recently, Braun et al. obtained an insulin-like growth factor 1-PEG (IGF1-PEG) conjugate for release in diseased tissue by using a combination of enzymatic and chemical bio-orthogonal coupling strategies. In this interesting example, mTGase was used for the ligation at the level of the N-terminal lysine of IGF1 to a PEG30 kDa modified protease-sensitive linker (Braun et al., 2018).

### Subtiligase

Subtiligase is a redesigned peptide ligase based on the modification of the active site of subtilisin. It was engineered by converting catalytic Ser221 to Cys, thereby increasing the ligase activity compared to amidase activity, and Residue Pro225 was converted to Ala to reduce steric assembling (Haridas et al., 2014). Subtiligase facilitates the ligation between a peptide C-terminal ester and a peptide N-terminal α-amine, without requiring a recognition motif at the termini of any reaction partners (Lin and He, 2018) (**Figure 3C**). The selective modification of the α-amine using subtiligase is a powerful approach in proteomics to enrich new N-termini arising from protease recognition and cleavage (Wiita et al., 2014), because 80% and 90% of wild-type eukaryotic proteins are acetylated at the N-terminal position (Polevoda and Sherman, 2003). This advantage could be exploited for the selective attachment of PEG-modified peptides as an innovative application to improve either the conjugation efficiency or the originality in the development of therapeutics.

#### Butelase 1

Butelase 1 is an enzyme isolated from the medicinal and ornamental plant *Clitoria ternatea,* which is a high-yielding asparagine/aspartate-specific cysteine ligase (Nguyen et al., 2014b) (**Figure 3D**). In spite of being C-terminal-specific for Asx, this enzyme accepts most N-terminal amino acids to mediate intermolecular peptide and protein ligation (Nguyen et al., 2016). Although it was recently discovered, *butelase 1* has been used for several purposes, such as protein modification and engineering, peptide/protein ligation and labeling, peptide/protein macrocyclization, and living-cell surface labeling (Lin and He, 2018). No work has yet reported *butelase 1* as being used for PEGylation reactions. However, some recent experiences with the enzyme, such as the method developed by Nguyen et al. for butelase-mediated ligation using thiodepsipeptides, have been applied in N-terminal labeling of ubiquitin and green fluorescent protein (GFP). The ligation yield of > 95% could be achieved for the model peptide and ubiquitin with a small substrate excess. This result anticipates a wide-ranging application and the perspectives of using *butelase 1* for N-terminal modification of peptides and proteins (Nguyen et al., 2015).

### Lipoid Acid Ligase

Lipoid acid ligase (LplA) is an alternative enzyme that has also been exploited for protein bioconjugation. This enzyme is able to recognize a specific LplA acceptor peptide (LAP) and catalyze the attachment of a lipoate moiety to a lysine residue in LAP (GFEIDKVWYDLDA) through an ATP-dependent reaction (Puthenveetil et al., 2009; Zhang et al., 2018) (**Figure 3E**). Regarding PEGylation, Plaks et al. used LplA for multisite clickable modification based on the incorporation of azide moieties in GFP at the N-terminal and two internal sites. Modification of the ligated azide groups with PEG, α--mannopyranoside and palmitic acid resulted in highly homogeneous populations of conjugates, being a potential approach, for instance, for site-specific multipoint protein PEGylation, among other modifications (Plaks et al., 2015). Additionally, other studies have been conducted using LplA-mediated enzymatic protein labeling followed by subsequent bio-orthogonal reactions (Hauke et al., 2014; Drake et al., 2016; Gray et al., 2016), allowing site-specific labeling of N- or C-terminus, even at the internal regions of a target protein.

There are other enzymes that have also been exploited for protein bioconjugation, including tubulin tyrosine ligase, which catalyzes the ATP-dependent addition of a tyrosine residue to the C-terminus of a-tubulin yielding a peptide bond (Schumacher et al., 2015; Zhang et al., 2018); N-myristoyltransferase, leading the transference of myristate from myristoyl-CoA to the N-terminal glycine of protein substrates, resulting in an amide linkage (Wright et al., 2010; Zhang et al., 2018); and biotin ligase, another ATP-dependent enzyme, catalyzes the conjugation of biotin derivatives onto proteins (Howarth and Ting, 2008; Fairhead and Howarth, 2015).

## Analytical Methods for Characterization of PEGylated Proteins

The evidence indicates that the use of PEG to improve the properties of biopharmaceuticals or diagnostic agents will increase. This is supported by the growing number of proposals in clinical evaluation each year. In order to achieve high-quality products, it is necessary to take into account the implementation of accurate methods for the analysis of some parameters that provide a higher level of characterization of the molecule under study. It is important to note that none of the techniques on their own allows for the most complete characterization of the PEGylated proteins, but in many cases the combination of these is necessary to obtain more accurate results. This section provides an overview of the most frequently used analytical methods for the characterization of PEGylated peptides and proteins.

### High-Performance Liquid Chromatography–Mass Spectrometry

High-performance liquid chromatography (HPLC) has been used for the separation and quantitation of free PEG and its PEGylated-protein form (PEG-conjugate). Some features of the PEGylated protein such as conjugate molecular weight, polymer mass distribution, or the degree and sites of PEGylation can be measured by HPLC methods. Lee et al., using SEC (size-exclusion chromatography) and RP-HPLC (reversed phase-high-performance liquid chromatography) mapping, assessed N-terminal PEGylated EGF, demonstrating the formation of a PEGylated macromolecule and that PEGylation occurred at the N-terminal position, respectively (Lee et al., 2003). Also, Brand et al. performed the separation of N-terminal PEGylated retargeted tissue factor tTF-NGR by using HPLC-based gel filtration, revealing pure elution fractions with the mono-PEGylated protein, which were represented by one clear band in SDS-PAGE and Western blotting (Brand et al., 2015). Although generally useful, the HPLC conditions and detection method must be improved for each compound based on the specific properties of the conjugated proteins.

To improve HPLC performance in the characterization of PEGylated proteins and to provide a more detailed characterization, the solution of coupling liquid chromatography to mass spectrometry was adopted. For decades, mass spectrometry (MS) has been the technique of choice for PEGylated protein characterization in terms of accurate average molecular weight and degree of PEGylation (Hutanu, 2014). A comparison of PEGylated and un-PEGylated counterparts by MS and peptide mapping is used to identify and quantify PEGylation sites and characterize impurities that occasionally go undetected by simpler techniques (Caserman et al., 2009). Collins et al. performed N-terminal amine PEGylation to stabilize oxytocin formulations for prolonged storage. Conjugation was confirmed by Matrix-Assisted Laser Desorption/Ionization Time-Of-Flight (MALDI-TOF) MS, where a clear shift in molecular weight was observed in the MALDI-TOF spectrum from the NHS ester polymer to the polymer-peptide conjugate (Collins et al., 2016). In another study conducted by Qin et al. following MALDI-TOF MS, PEG modification sites were determined through comparative analysis of peptide mapping between rhGH (recombinant human growth hormone) and PEG-rhGH. The use of MS makes it possible to discriminate positional isomers, with PEGylation sites potentially located at the N-terminus and nine lysine residues of rhGH (Qin et al., 2017). However, the exact determination of the PEG attachment site(s) continues to be highly challenging, especially in a mixture composed of products with differing degrees of PEGylation (Gerislioglu et al., 2018). On the other hand, ESI-TOF has overcome some disadvantages related to polydispersion and the overlapping protein charge pattern of PEGylated proteins (Forstenlehner et al., 2014). Furthermore, ESI-MS is preferred to MALDI due to automated workflow and reduced sample preparation time (Hutanu, 2014). Several studies have reported applying the approach of the line-up of liquid chromatography to MS (LC-MS) for the sensitive quantitation of free PEG in biological fluid samples (Pelham et al., 2008; Yin et al., 2017) or tissues (Gong et al., 2014), as well as clear detection and identification of the positional isomers formed upon PEGylation (Gerislioglu et al., 2018; Shekhawat et al., 2019), obtaining significant structural information in a heterogeneous sample of PEGylated proteins (Mero et al., 2016; Muneeruddin et al., 2017), among others.

### Dynamic Light Scattering

Dynamic light scattering (DLS) is an additional technique also convenient for the molecular weight evaluation of PEGylated proteins, as it can measure the molecular radii of the samples (Kusterle et al., 2008; Gokarn et al., 2012), and discriminate between linear and branched PEGs (Wan et al., 2017). This method, among others, was used by Vernet et al. in 2016 to assess the first large-scale study with the site-specific mono-PEGylation of 15 different proteins and characterization of 61 entities in total (Vernet et al., 2016). In addition, Khameneh et al. conducted a study in which site-specific PEGylated hGH was prepared by using microbial transglutaminase. Physicochemical properties, size and zeta potentials of native and PEGylated hGH, were evaluated by DLS, indicating that the size and zeta potentials of the protein were increased and decreased respectively by PEGylation, enhancing the stability of the protein (Khameneh et al., 2016). Recently, Meneguetti et al. applied DLS for the characterization of a novel N-terminal PEGylated asparaginase, showing that the PEGylation of ASNase caused an increase in the hydrodynamic diameter of the protein related to the increase in the amount of PEG attached to the protein (Meneguetti et al., 2019). The DLS approach has been used in the characterization not only of PEGylated proteins, but also of PEGylated organic nanotubes, revealing that PEGylation dramatically improves the dispersibility of the nanotubes in saline buffer (Ding et al., 2014). Despite its wide use in the characterization of the hydrodynamic radius of PEGylated proteins, this methodology presents certain disadvantages in its application, such as the presence of large particles that can also be detected during the analysis; low resolution when the populations are close in size or a highly polydispersed sample; light absorption by the dispersant can interfere with detection because of their viscosity as well as the density of the particles. These are important parameters to take into account when carrying out this type of analysis.

### Nuclear Magnetic Resonance

^1^H nuclear magnetic resonance (NMR) spectroscopy is useful to quantify PEGylated species in complex biological fluids with advantages of time and simplicity in the sample preparation (Alvares et al., 2016). The application of this technique for the structural characterization of conjugates with PEG (Kiss et al., 2018) has been being useful in the quantitative determination of the degree of PEGylation (Zaghmi et al., 2019), the assessment of the higher-order structure of PEGylated therapeutic proteins (Cattani et al., 2015; Hodgson and Aubin, 2017) or even the behavior of free PEG in serum samples (Khandelwal et al., 2019). More recently, solid state NMR has been used for the structural characterization of large PEGylated proteins such as asparaginase (Giuntini et al., 2017; Cerofolini et al., 2019). The combination of NMR with other techniques such as LC-MS/MS has enabled the accurate quantification of isobaric glycan structures, even in the picomolar order (Wiegandt and Meyer, 2014), an approach that could be used for a better characterization of high complexity PEGylated molecules.

### Immunoenzymatic Assays

Enzyme-linked immunosorbent assay (ELISA) is a powerful tool for measuring the concentration of PEGylated proteins in serum samples (Wang et al., 2012). This technique permits the study of the effects of PEGylation in protein immunogenicity as well as the anti-PEG immune response (Wan et al., 2017). While direct ELISA has the advantage of lacking only one specific antibody for compound detection, it cannot distinguish between PEGylated and unPEGylated proteins (Cao et al., 2009). On the other hand, sandwich or indirect ELISA employs two antibodies: one to capture the analyte on a solid surface and a second to determine the concentration of the captured analyte (Gan and Patel, 2013). Bruno et al. used a quantitative sandwich ELISA to analyze the pharmacokinetics of Pegasys and PEG-Intron using two mouse monoclonal antihuman IFN-α antibodies that recognize different epitopes of IFN-α (Bruno et al., 2004), and a similar ELISA was used for the measurement of Neulasta^®^ (Roskos et al., 2006) and Mircera^®^ (Macdougall et al., 2006). Su et al. produced second-generation monoclonal antibodies attached to PEG (AGP4/IgM and 3.3/IgG) that also bind to the repeating subunits of the PEG backbone, but with greater affinity than those of first-generation AGP3 and E11 (Su et al., 2010). Since then, they have produced a range of specific anti-PEG IgG and IgM monoclonal antibodies for use in ELISA, FACs, IHC, and flow cytometry, which can be found under anti-PEG in the Institute of Biomedical Sciences at Academia Sinica, Taiwan.

### Bioinformatics Methods

With the advent of the era of bioinformatics, computational methods have been effectively employed for an easier designing, engineering, and characterization of proteins, which supports experimental methodologies and, in many cases, saves time and materials. At present, computational analysis is highly recommended to select the proper position on the protein for site-selective PEGylation (Rouhani et al., 2018). In 2013 Mu et al. conducted a bioinformatics study in which four forms of PEGylated staphylokinase obtained by site-specific conjugation of PEG to the N- and C-termini of SK, respectively, were structurally evaluated to provide greater molecular insight into the interaction between the PEGylated protein and its receptor (Mu et al., 2013). The results suggested that the PEG polymer wraps around the protein providing steric shield, and this effect depends on the PEG chain length and PEGylation site of the protein (Rouhani et al., 2018b). Also, Mirzaei et al. (2016) applied computational and non-glycosylated systems to define an artless methodology for site-selective (cysteine) PEGylation of erythropoietin analogs. The results showed that using an *in silico* approach together with the experimental methodologies can be a strategy to optimize the parameters of PEGylation (Mirzaei et al., 2016). Recently, Xu et al. (2018) used interferon (IFN) as a representative model system to characterize the molecular-level changes in IFN introduced by several degrees of PEGylation through molecular dynamics simulations. The simulations generated molecular evidence directly linked to improved protein stability, bioavailability, retention time, as well as the decrease in protein bioactivity with PEG conjugates, providing an important computational approach in the improvement of PEGylated protein drug conjugates and their clinical performance (Xu et al., 2018a). However, and in spite of the advances obtained in this field, there are still some drawbacks that must be solved, such as the computational cost in terms of infrastructure, and many times, it could be hard to explain what the biological or clinical meaning of features identified using bioinformatics analysis.

## Recent Approaches in the Site-Selective Conjugation of Proteins

The chemistry of natural amino acids has been a highly exploited approach in the bioconjugation of proteins. However, there is often poor control over the site and various modifications and incompatibilities with complex mixtures or living systems (Reddington and Howarth, 2015). Since the manipulation of proteins is at the core of biochemical research, the search for new strategies in efficient and specific bioconjugation has been an objective developed by the scientific community through protein engineering. These strategies include for example the SpyTag/SpyCatcher system.

### The Spytag/Spycatcher System

The *SpyTag/SpyCatcher* system allows the specific and covalent conjugation of proteins through two short polypeptide tags (Zakeri et al., 2012). The larger partner, the SpyCatcher, adopts an immunoglobulin-like conformation that specifically binds the SpyTag (γ-carbon of Asp-117), leading the formation of an extremely resistant intermolecular bond between two amino acid side chains (Gilbert et al., 2017). In this extremely fast method, no exogenous enzymes need to be added or removed (Fisher et al., 2017) and despite its recent description, this system has already been used in the production of synthetic vaccines (Brune et al., 2016), thermo-stable enzymes (Schoene et al., 2016; Wang et al., 2016), and other applications (Fierer et al., 2014; Dovala et al., 2016; Lakshmanan et al., 2016). Take advantage of this system, Gilbert et al. described how the XynA enzyme was genetically encoded to covalently conjugate in culture media, providing a novel and flexible strategy for protein conjugation exploiting the substantial advantages of extracellular self-assembly (Gilbert et al., 2017). Recently, Cayetano-Cruz et al. published a study in which the α-glucosidase Ima1p enzyme of *Saccharomyces cerevisiae* was attached to the surface of virus-like particles (VLPs) of parvovirus B19 using the SpyTag/SpyCatcher system. This approach made it possible to obtain a more thermostable enzyme and the modified VLPs were also able to act on glycogen. Hence, these particles may be developed in the future as part of the therapy for the treatment of diseases caused by defects in the human acid α-glucosidase (Cayetano-Cruz et al., 2018). SpyCatcher is large and may be difficult to attach to polymers; therefore, the final product contains a large SpyCatcher protein sequence (Zakeri et al., 2012). It could be a reason why no study to date has been reported using this system to modify proteins with PEGs. However, this is a promising mechanism to create PEGylated proteins, taking advantage of the fact that SpyTag can be placed at the N-terminus, at the C-terminus and at the internal positions of a protein (Zakeri et al., 2012), and previously bound, for instance, to the polymers (PEG) being conjugated.

### Ring Opening Polymerization

Ring opening polymerization (ROP) is a reaction, in which the terminal end of a polymer chain acts as a reactive center where additional cyclic monomers can react by opening its ring system, forming a longer polymer chain (Jenkins et al., 1996) with the occurrence of two main reactions: initiation and growth (Penczek and Pretula, 2016). In 2013, Spears et al. used the approach of ROP for first time for the *in situ* controlled branching of polyglycidol and formation of BSA-glycidol bioconjugates with “PEG-like” arms (Spears et al., 2013; Qi and Chilkoti, 2015). Since then, ROP has been used as a methodology to modify various molecules as well as to obtain different varieties of polymers. Ma et al. prepared a cross-linked fluorescent polymer through ROP and performed a subsequent ring opening PEGylation with 4-arm PEG-amine, yielding polymeric nanoparticles in aqueous solution with hydrophilic PEG groups covered at the surface (Ma et al., 2015). Also, Tian et al. (2018) developed smart polymeric materials based on biomimetic PEGylated polypeptoids by combining ring-opening polymerization and a post-modification strategy (Tian et al., 2018). Furthermore, the usefulness of this approach has also been established in the preparation of PEGylated and fluorescent nanoprobes for biomedical applications (Wan et al., 2015; Xu et al., 2018b) and the development of polymeric gene vectors with high transfection efficiency and improved biocompatibility (Xiao et al., 2018). All this demonstrates the potential that ROP could have in the design of PEGylated proteins of biopharmaceutical interest or other molecules used in the diagnosis of different diseases.

### Click Chemistry

Click chemistry is another method widely used for PEG attachment to proteins for different purposes (Jølck et al., 2010; Leung et al., 2012; Li et al., 2012; Xu et al., 2015; Huang et al., 2018; Lou et al., 2018). Here, azide and alkyne groups react selectively with each other in the presence of Cu^1+^ as the catalyst (Rostovtsev et al., 2002) through the initial reaction of reduced thiols with a maleimide compound containing a click-reactive alkyne moiety. Then, a large PEG molecule containing a complementary click-reactive azide moiety is selectively conjugated to the click-tagged thiols (van Leeuwen et al., 2017). This method is versatile, fast and simple to use, easy to purify, site-specific, and gives high product yields (Hein et al., 2008); however, its drawback is related to the toxicity of copper, even in small amounts. This could limit the development of pharmaceuticals using this methodology; as a result, PEGylation *via* copper-free click reaction has gained more attention nowadays (Debets et al., 2010; Koo et al., 2012; Lou et al., 2018). The reaction conditions are extremely mild and do not cause protein denaturation, nor are any metals, reducing agents or ligands required.

### Non-Covalent PEGylation

Non-covalent PEGylation is an innovative approach in which a chemical reaction between protein and PEG is avoided (Reichert and Borchard, 2016). It is based on the mechanisms of hydrophobic interactions (Mueller et al., 2011a; Mueller et al., 2011b; Mueller et al., 2012), ionic interactions (Khondee et al., 2011), protein polyelectrolyte complex (Kurinomaru and Shiraki, 2015; Kurinomaru et al., 2017), or chelation (Mero et al., 2011). The main advantage of this technique is that it eliminates a potential loss of product due to additional purification processes (Reichert and Borchard, 2016). However, the release of the protein during storage is an important shortcoming for this approach (Santos et al., 2018).

## Concluding Remarks

The covalent attachment of peptides and proteins to polyethylene glycol remains a preferred method for modifying the pharmacokinetic and immunological properties of therapeutic molecules, supported not only by the introduction of PEGylated drugs on the market but also by the increasing number of currently ongoing clinical studies. The chemical versatility of polyethylene glycol derivatives enables the synthesis of various PEGylated protein structures, with a trend to target-specific amino acid residues located at the terminal ends (N or C-terminus) of the peptides or protein of interest, which contributes to obtaining homogeneous and well-defined conjugates. These site-selective modifications must preserve the biological activity of the PEGylated molecule. As part of the development of the science of PEGylation, new methods continue to be implemented based on new approaches, as well as faster and more efficient techniques, such as enzymatic ligation or the development of bio-orthogonal chemistry. As the number and location of PEG chains attached to a protein can affect its activity, it is critical to uncover these important structural details. Thus, strong analytical methods must be developed, allowing for a qualitative and quantitative characterization with a greater degree of robustness and accuracy. In this sense, the computational tools (predictive models based on molecular dynamics) are a great help in clarifying interactions, binding sites or stability of PEGylated proteins in the unending search for and design of new, more effective biopharmaceuticals.

## Author Contributions

LB and CR-Y: writing of the topic related to the chemical reactions of site-specific pegylation. JBL and ML-E: writing of the topic related to enzymatic pegylation. BE and RC: writing of the topic related to the characterization of pegylated proteins. AP and JF: critical revisions and corrections of the manuscript.

## Conflict of Interest

The authors declare that the research was conducted in the absence of any commercial or financial relationships that could be construed as a potential conflict of interest.

## References

[B1] Abbina S. Parambath A. (2018). “PEGylation and its alternatives: A summary,” in Engineering of Biomaterials for Drug Delivery Systems: Beyond Polyethylene Glycol. Woodhead Publishing 363–376.

[B2] Adrio J. L. Demain A. L. (2014). Microbial enzymes: tools for biotechnological processes. Biomolecules 4 (1), 117–139. 10.3390/biom4010117 24970208 PMC4030981

[B3] Agrawal V. Hee Woo J. Borthakur G. Kantarjian H. Frankel E. A. (2013). Red blood cell-encapsulated L-Asparaginase: potential therapy of patients with Asparagine Synthetase deficient acute myeloid leukemia. Protein Pept. Lett. 20, 392–402. 10.2174/092986613805290426 23016580

[B4] Alconcel S. N. S. Baas A. S. Maynard H. D. (2011). FDA-approved poly(ethylene glycol)-protein conjugate drugs. Polym. Chem. 2, 1442–1448. 10.1039/c1py00034a

[B5] Alvares R. D. A. Hasabnis A. Prosser R. S. Macdonald P. M. (2016). Quantitative detection of PEGylated biomacromolecules in biological fluids by NMR. Anal. Chem. 88 (7), 3730–3738. 10.1021/acs.analchem.5b04565 26927487

[B6] Ameringer T. Ercole F. Tsang K. M. Coad B. R. Hou X. Rodda A. (2013). Surface grafting of electrospun fibers using ATRP and RAFT for the control of biointerfacial interactions. Biointerphases 8 (1), 16. 10.1186/1559-4106-8-16 24706129

[B7] Berberich J. A. Yang L. W. Madura J. Bahar I. Russell A. J. (2005). A stable three-enzyme creatinine biosensor. 1. Impact of structure, function and environment on PEGylated and immobilized sarcosine oxidase. Acta Biomater. 10.1016/j.actbio.2004.11.006 16701794

[B8] Boutureira O. Bernardes G. J. L. (2015). Advances in chemical protein modification. Chem. Rev. 115 (5), 2174–2195. 10.1021/cr500399p 25700113

[B9] Brand C. Fröhlich M. Ring J. Schliemann C. Kessler T. Mantke V. (2015). Tumor growth inhibition via occlusion of tumor vasculature induced by N-terminally PEGylated retargeted tissue factor tTF-NGR. Mol. Pharm. 12 (10), 3749–3758. 10.1021/acs.molpharmaceut.5b00508 26310827

[B10] Braun A. C. Gutmann M. Mueller T. D. Lühmann T. Meinel L. (2018). Bioresponsive release of insulin-like growth factor-I from its PEGylated conjugate. J. Control. Release. 279, 17–28. 10.1016/j.jconrel.2018.04.009 29634992

[B11] Brune K. D. Leneghan D. B. Brian I. J. Ishizuka A. S. Bachmann M. F. Draper S. J. (2016). Plug-and-Display: decoration of Virus-Like Particles via isopeptide bonds for modular immunization. Sci. Rep. 6, 19234. 10.1038/srep19234 26781591 PMC4725971

[B12] Bruno R. Sacchi P. Ciappina V. Zochetti C. Patruno S. Maiocchi L. (2004). Viral dynamics and pharmacokinetics of peginterferon alpha-2a and peginterferon alpha-2b in naive patients with chronic hepatitis C: A randomized, controlled study. Antivir. Ther. 9 (4), 369–376. 10.1111/j.1365-2036.2007.03392.x 15456079

[B13] Busch G. K. Tate E. W. Gaffney P. R. J. Rosivatz E. Woscholski R. Leatherbarrow R. J. (2008). Specific N-terminal protein labelling: Use of FMDV 3Cproprotease and native chemical ligation. Chem. Commun. (29), 3369–3371. 10.1039/b806727a 18633492

[B14] Cabrales P. Tsai A. G. Ananda K. Acharya S. A. Intaglietta M. (2008). Volume resuscitation from hemorrhagic shock with albumin and hexaPEGylated human serum albumin. Resuscitation 79 (1), 139–146. 10.1016/j.resuscitation.2008.04.020 18621463 PMC2758307

[B15] Cano F. Alarcon C. Azocar M. Lizama C. Maria Lillo A. Delucchi A. (2011). Continuous EPO receptor activator therapy of anemia in children under peritoneal dialysis. Pediatr. Nephrol. 26 (8), 1303–1310. 10.1007/s00467-011-1846-5 21416403

[B16] Cao J. Du Y. Tian H. Gao X. D. Yao W. B. (2009). Quantitative determination of pegylated consensus interferon in rhesus monkey serum using a competitive enzyme-linked immunosorbent assay. Immunopharmacol. Immunotoxicol. 31 (4), 543–549. 10.3109/08923970902814111 19874220

[B17] Caserman S. Kusterle M. Kunstelj M Milunović T Schiefermeier M Jevsevar S (2009). Correlations between *in vitro* potency of polyethylene glycol-protein conjugates and their chromatographic behavior. Anal. Biochem. 389 (1), 27–31. 10.1016/j.ab.2009.03.023 19306838

[B18] Cattani G. Vogeley L. Crowley P. B. (2015). Structure of a PEGylated protein reveals a highly porous double-helical assembly. Nat. Chem. 7 (10), 823–828. 10.1038/nchem.2342 26391082

[B19] Cayetano-Cruz M. Coffeen C. F. Valadez-García J. Montiel C. Bustos-Jaimes I. (2018). Decoration of virus-like particles with an enzymatic activity of biomedical interest. Virus Res. 255, 1–9. 10.1016/j.virusres.2018.06.014 29964063

[B20] Cerofolini L. Giuntini S. Carlon A. Ravera E. Calderone V. Fragai M. (2019). Characterization of PEGylated Asparaginase: new opportunities from NMR analysis of large PEGylated therapeutics. Chem. Eur. J. 25 (8), 1984–1991. 10.1002/chem.201804488 30462348

[B21] Cesaro S. Nesi F. Tridello G. Abate M. Panizzolo I. S. Balter R. (2013). A randomized, non-inferiority study comparing efficacy and safety of a single dose of pegfilgrastim versus daily filgrastim in pediatric patients after autologous peripheral blood stem cell transplant. PloS One 8 (1), e53252. 10.1371/journal.pone.0053252 23308174 PMC3538773

[B22] Chan W. K. Ho C. M. Wong M. K. Che C. M. (2006). Oxidative amide synthesis and N-terminal α-amino group ligation of peptides in aqueous medium. J. Am. Chem. Soc. 128 (46), 14796–14797. 10.1021/ja064479s 17105276

[B23] Chan A. O.-Y. Ho C.-M. Chong H.-C. Leung Y.-C. Huang J.-S. Wong M.-K. (2012). Modification of N-terminal α-amino groups of peptides and proteins using ketenes. J. Am. Chem. Soc. 134 (5), 2589–2598. 10.1021/ja208009r 22288779

[B24] Chen H. He S. (2015). PLA-PEG coated multifunctional imaging probe for targeted drug delivery. Mol. Pharm. 12 (6), 1885–1898. 10.1021/mp500512z 25871882

[B25] Chen D. Disotuar M. M. Xiong X. Wang Y. Chou D. H.-C. (2017). Selective N-terminal functionalization of native peptides and proteins. Chem. Sci. 8 (4), 2717–2722. 10.1039/C6SC04744K 28553506 PMC5426342

[B26] Chen W. Gao P. Jiang H. Cui Z. (2019). A novel reactive dyeing method for silk fibroin with aromatic primary amine-containing dyes based on the Mannich reaction. Dye. Pigment. 168, 300–310. 10.1016/j.dyepig.2019.04.061

[B27] Chiu K. Agoubi L. L. Lee I. Limpar M. T. Lowe J. W. Goh S. L. (2010). Effects of polymer molecular weight on the size, activity, and stability of PEG-functionalized trypsin. Biomacromolecules 11 (12), 3688–3692. 10.1021/bm1006954 20979350

[B28] Collins J. Kempe K. Wilson P. Blindauer C. McIntosh M. P. Davis T. P. (2016). Stability enhancing n-terminal pegylation of oxytocin exploiting different polymer architectures and conjugation approaches. Biomacromolecules 17 (8), 2755–2766. 10.1021/acs.biomac.6b00919 27419537

[B29] D’souza A. A. Shegokar R. (2016). Polyethylene glycol (PEG): a versatile polymer for pharmaceutical applications. Expert Opin. Drug Deliv. 10.1080/17425247.2016.1182485 27116988

[B30] Da Silva Freitas D. Mero A. Pasut G. (2013). Chemical and enzymatic site specific pegylation of hGH. Bioconjug. Chem. 24 (3), 456–463. 10.1021/bc300594y 23432141

[B31] David Y. Vila-Perelló M. Verma S. Muir T. W. (2015). Chemical tagging and customizing of cellular chromatin states using ultrafast trans-splicing inteins. Nat. Chem. 7 (5), 394–402. 10.1038/nchem.2224 25901817 PMC4617616

[B32] Debets M. F. Van Berkel S. S. Schoffelen S. Rutjes F. P. J. T. Van Hest J. C. M. Van Delft F. L. (2010). Aza-dibenzocyclooctynes for fast and efficient enzyme PEGylation via copper-free (3 + 2) cycloaddition. Chem. Commun. 46 (1), 97–99. 10.1039/b917797c 20024305

[B33] Ding W. Minamikawa H. Kameta N. Shimizu T. Masuda M. (2014). Effects of PEGylation on the physicochemical properties and in vivo distribution of organic nanotubes. Int. J. Nanomedicine. 9, 5811–5823. 10.2147/IJN.S75604 25540582 PMC4270402

[B34] Dovala D. Sawyer W. S. Rath C. M. Metzger L. E. (2016). Rapid analysis of protein expression and solubility with the SpyTag-SpyCatcher system. Protein Expr. Purif. 117, 44–51. 10.1016/j.pep.2015.09.021 26405011

[B35] Dozier J. K. Distefano M. D. (2015). Site-specific pegylation of therapeutic proteins. Int. J. Mol. Sci. 16 (10), 25831–25864. 10.3390/ijms161025831 26516849 PMC4632829

[B36] Drake C. R. Sevillano N. Truillet C. Craik C. S. VanBrocklin H. F. Evans M. J. (2016). Site-specific radiofluorination of biomolecules with 8-[18F]-fluorooctanoic acid catalyzed by lipoic acid ligase. ACS Chem. Biol. 11 (6), 1587–1594. 10.1021/acschembio.6b00172 27008570 PMC5712215

[B37] Erfani-Jabarian L. Dinarvand R. Rouini M. R. Atyabi F. Amini M. Mohammadhosseini N. (2012). PEGylation of octreotide using an α,β-unsaturated-β′-mono-sulfone functionalized PEG reagent. Iran. J. Pharm. Res. 11 (3), 747–753. 24250501 PMC3813111

[B38] Ettinger A. R. (1995). Pegaspargase (Oncaspar). J. Pediatr. Oncol. Nurs. 12 (1), 46–48. 10.1177/104345429501200110 7893462

[B39] Fairhead M. Howarth M. (2015). “Site-Specific biotinylation of purified proteins using BirA,” in Site-Specific Protein Labeling: Methods Mol. Biol. 1266, 171–84. 10.1007/978-1-4939-2272-7_12 PMC4304673 25560075

[B40] Fierer J. O. Veggiani G. Howarth M. (2014). SpyLigase peptide–peptide ligation polymerizes affibodies to enhance magnetic cancer cell capture. Proc. Natl. Acad. Sci. 111 (13), E1176–E1181. 10.1073/pnas.1315776111 24639550 PMC3977242

[B41] Fisher S. A. Baker A. E. G. Shoichet M. S. (2017). Designing Peptide and Protein Modified Hydrogels: Selecting the Optimal Conjugation Strategy. J. Am. Chem. Soc. 139 (22), 7416–7427. 10.1021/jacs.7b00513 28481537

[B42] Forstenlehner I. C. Holzmann J. Scheffler K. Wieder W. Toll H. Huber C. G. (2014). A direct-infusion-and HPLC-ESI-orbitrap-MS approach for the characterization of intact pegylated proteins. Anal. Chem. 86 (1), 826–834. 10.1021/ac403390y 24308604

[B43] Foser S. Schacher A. Weyer K. A. Brugger D. Dietel E. Marti S. (2003). Isolation, structural characterization, and antiviral activity of positional isomers of monopegylated interferon α-2a (PEGASYS). Protein Expr. Purif. 30 (1), 78–87. 10.1016/S1046-5928(03)00055-X 12821324

[B44] Gaertner H. F. Offord R. E. (1996). Site-specific attachment of functionalized poly(ethylene glycol) to the amino terminus of proteins. Bioconjug. Chem. 7 (1), 38–44. 10.1021/bc950074d 8741989

[B45] Gan S. D. Patel K. R. (2013). Enzyme immunoassay and enzyme-linked immunosorbent assay. J. Invest. Dermatol. 133 (9), e12. 10.1038/jid.2013.287 23949770

[B46] Ganesan P. Soundararajan R. Shanmugam U. Ramu V. (2015). Development, characterization and solubility enhancement of comparative dissolution study of second generation of solid dispersions and microspheres for poorly water soluble drug. Asian J. Pharm. Sci. 10 (5), 433–441. 10.1016/j.ajps.2015.05.001

[B47] Gauthier M. A. Klok H. A. (2008). Peptide/protein-polymer conjugates: Synthetic strategies and design concepts. Chem. Commun. (23), 2591–2611. 10.1039/b719689j 18535687

[B48] Gentle I. E. De Souza D. P. Baca M. (2004). Direct production of proteins with N-terminal cysteine for site-specific conjugation. *Bioconjug. Chem.* . 15 (3), 658–663. 10.1021/bc049965o 15149194

[B49] Gerislioglu S. Adams S. R. Wesdemiotis C. (2018). Characterization of singly and multiply PEGylated insulin isomers by reversed-phase ultra-performance liquid chromatography interfaced with ion mobility mass spectrometry. Anal. Chim. Acta. 1004, 58–66. 10.1016/j.aca.2017.12.009 29329709

[B50] Gilbert C. Howarth M. Harwood C. R. Ellis T. (2017). Extracellular self-assembly of functional and Tunable protein conjugates from Bacillus subtilis. ACS Synth. Biol. 6 (6), 957–967. 10.1021/acssynbio.6b00292 28230977

[B51] Gilmore J. M. Scheck R. A. Esser-Kahn A. P. Joshi N. S. Francis M. B. (2006). N-terminal protein modification through a biomimetic transamination reaction. Angew. Chemie Int. Ed. 45 (32), 5307–5311. 10.1002/anie.200600368 16847857

[B52] Giuntini S. Balducci E. Cerofolini L. Ravera E. Fragai M. Berti F. (2017). Characterization of the conjugation pattern in large polysaccharide–protein conjugates by NMR spectroscopy. Angew. Chemie Int. Ed. 56 (47), 14997–15001. 10.1002/anie.201709274 PMC5813213 29024352

[B53] Gody G. Barbey R. Danial M. Perrier S. (2015). Ultrafast RAFT polymerization: Multiblock copolymers within minutes. Polym. Chem. 6, 1502–1511. 10.1039/c4py01251h

[B54] Gokarn Y. R. McLean M. Laue T. M. (2012). Effect of PEGylation on protein hydrodynamics. Mol. Pharm. 9 (4), 762–773. 10.1021/mp200470c 22353017

[B55] Gong J. Gu X. Achanzar W. E. Chadwick K. D. Gan J. Brock B. J. (2014). Quantitative analysis of polyethylene glycol (PEG) and PEGylated proteins in animal tissues by LC-MS/MS coupled with in-source CID. Anal. Chem. 86 (15), 7642–7649. 10.1021/ac501507g 25003239

[B56] Gray M. A. Tao R. N. Deporter S. M. Spiegel D. A. McNaughton B. R. (2016). A nanobody activation immunotherapeutic that selectively destroys HER2-positive breast cancer cells. ChemBioChem. 17 (2), 155–158. 10.1002/cbic.201500591 26556305 PMC5199233

[B57] Griffin M. Casadio R. Bergamini C. M. (2002). Transglutaminases: nature’s biological glues. Biochem. J. 368 (Pt 2), 377–379. 10.1042/bj20021234 12366374 PMC1223021

[B58] Gunnoo S. B. Madder A. (2016). Chemical protein modification through cysteine. ChemBioChem. 17 (7), 529–553. 10.1002/cbic.201500667 26789551

[B59] Hamidi M. Azadi A. Rafiei P. (2006). Pharmacokinetic consequences of pegylation. Drug Deliv. 13, 399–409. 10.1080/10717540z600814402 17002967

[B60] Haridas V. Sadanandan S. Dheepthi N. U. (2014). Sortase-based bio-organic strategies for macromolecular synthesis. ChemBioChem. 15 (13), 1857–67. 10.1002/cbic.201402013 25111709

[B61] Harvey L. Arnold B. Lawrence Z. Paul M. David B. James D. (2000). Molecular Cell Biology, 4th edition (New York: W. H. Freeman)

[B62] Hauke S. Best M. Schmidt T. T. Baalmann M. Krause A. Wombacher R. (2014). Two-step protein labeling utilizing lipoic acid ligase and sonogashira cross-coupling. Bioconjug. Chem. 25 (9), 1632–1637. 10.1021/bc500349h 25152073

[B63] Hein C. D. Liu X. M. Wang D. (2008). Click chemistry, a powerful tool for pharmaceutical sciences. Pharm. Res. 25 (10), 2216–2230. 10.1007/s11095-008-9616-1 18509602 PMC2562613

[B64] Hodgson D. J. Aubin Y. (2017). Assessment of the structure of pegylated-recombinant protein therapeutics by the NMR fingerprint assay. J. Pharm. Biomed. Anal. 138, 351–356. 10.1016/j.jpba.2017.01.058 28254519

[B65] Howarth M. Ting A. Y. (2008). Imaging proteins in live mammalian cells with biotin ligase and monovalent streptavidin. Nat. Protoc. 3 (3), 534–545. 10.1038/nprot.2008.20 18323822 PMC2671200

[B66] Huang J. Qin H. Sun Z. Huang G. Mao J. Cheng K. (2015). A peptide N-terminal protection strategy for comprehensive glycoproteome analysis using hydrazide chemistry based method. Sci. Rep. 5, 10164. 10.1038/srep10164 25959593 PMC4426672

[B67] Huang H. Liu M. Tuo X. Chen J. Mao L. Wen Y. (2018). One-step fabrication of PEGylated fluorescent nanodiamonds through the thiol-ene click reaction and their potential for biological imaging. Appl. Surf. Sci. 439, 1143–1151. 10.1016/j.apsusc.2017.12.233

[B68] Hutanu D. (2014). Trends in characterization of PEGylated proteins by mass spectrometry. Mod. Chem. Appl. 2, 2. 10.4172/2329-6798.1000128

[B69] Jenkins A. D. Kratochvíl P. Stepto R. F. T. Suter U. W. (1996). Glossary of basic terms in polymer science (IUPAC Recommendations 1996). Pure Appl. Chem. 68 (12), 2287–2311. 10.1351/pac199668122287

[B70] Jevševar S. Kunstelj M. Porekar V. G. (2010). PEGylation of therapeutic proteins. Biotechnol. J. 5 (1), 113–128. 10.1002/biot.200900218 20069580

[B71] Johnson E. C. B. Kent S. B. H. (2006). Insights into the mechanism and catalysis of the native chemical ligation reaction. J. Am. Chem. Soc. 128(20), 6640–6646. 10.1021/ja058344i 16704265

[B72] Jølck R. I. Berg R. H. Andresen T. L. (2010). Solid-phase synthesis of PEGylated lipopeptides using click chemistry. Bioconjug. Chem. 5, 807–810. 10.1021/bc100002a 20481501

[B73] Jones M. W. Mantovani G. Blindauer C. A. Ryan S. M. Wang X. Brayden D. J. (2012). Direct peptide bioconjugation/PEGylation at tyrosine with linear and branched polymeric diazonium salts. J. Am. Chem. Soc. 134 (17), 7406–7413. 10.1021/ja211855q 22494012

[B74] Khameneh B. Saberi M. R. Hassanzadeh-Khayyat M. Mohammadpanah H. Ghandadi M. Iranshahi M. (2016). Evaluation of physicochemical and stability properties of human growth hormone upon enzymatic PEGylation. J. Appl. Biomed. 14, 257–264. 10.1016/j.jab.2016.06.002

[B75] Khandelwal P. Zhang L. Chimalakonda A. Caceres-Cortes J. Huang C. Marathe P. (2019). Pharmacokinetics of 40 kDa PEG in rodents using high-field NMR spectroscopy. J. Pharm. Biomed. Anal. 10.1016/j.jpba.2019.03.066 30959317

[B76] Khondee S. Olsen C. M. Zeng Y. Middaugh C. R. Berkland C. (2011). Noncovalent PEGylation by polyanion complexation as a means to stabilize keratinocyte growth factor-2 (KGF-2). Biomacromolecules 12 (11), 3880–3894. 10.1021/bm2007967 21954860

[B77] Kim T. Y. Seo H. D. Lee J. Kang J. A. Kim W. S. Kim H. M. (2018). A dimeric form of a small-sized protein binder exhibits enhanced anti-tumor activity through prolonged blood circulation. J. Control. Release 279, 282–291. 10.1016/j.jconrel.2018.04.039 29698702

[B78] Kinstler O. B. Brems D. N. Lauren S. L. Paige A. G. Hamburger J. B. Treuheit M. J. (1996). Characterization and stability of N-terminally PEGylated rhG-CSF. Pharm. Res. 13 (7), 996–1002. 10.1023/A:1016042220817 8842035

[B79] Kiss R. Fizil Á. Szántay C. (2018). What NMR can do in the biopharmaceutical industry. J. Pharm. Biomed. Anal. 147, 367–377. 10.1016/j.jpba.2017.07.004 28760370

[B80] Kochendoerfer G. (2003). Chemical and biological properties of polymer-modified proteins. Expert Opin. Biol. Ther. 3 (8), 1253–1261. 10.1517/14712598.3.8.1253 14640951

[B81] Koniev O. Wagner A. (2015). Developments and recent advancements in the field of endogenous amino acid selective bond forming reactions for bioconjugation. Chem. Soc Rev. 44, 5495–5551. 10.1039/c5cs00048c 26000775

[B82] Koo H. Lee S. Na J. H. Kim S. H. Hahn S. K. Choi K. (2012). Bioorthogonal copper-free click chemistry inVivo for tumor-targeted delivery of nanoparticles. Angew. Chemie Int. Ed. 51 (47), 11836–11840. 10.1002/anie.201206703 23081905

[B83] Krall N. Da Cruz F. P. Boutureira O. Bernardes G. J. L. (2016). Site-selective protein-modification chemistry for basic biology and drug development. Nat. Chem. 8 (2), 103–113. 10.1038/nchem.2393 26791892

[B84] Kubetzko S. (2005). Protein PEGylation decreases observed target association rates via a dual blocking mechanism. Mol. Pharmacol. 68 (5), 1439–1454. 10.1124/mol.105.014910 16099846

[B85] Kung K. K. Y. Wong K. F. Leung K. C. Wong M. K. (2013). N-terminal α-amino group modification of peptides by an oxime formation-exchange reaction sequence. Chem. Commun. 49 (61), 6888–6890. 10.1039/c3cc42261e 23792565

[B86] Kurinomaru T. Shiraki K. (2015). Noncovalent PEGylation of L-asparaginase using PEGylated polyelectrolyte. J. Pharm. Sci. 104 (2), 587–592. 10.1002/jps.24217 25354692

[B87] Kurinomaru T. Kuwada K. Tomita S. Kameda T. Shiraki K. (2017). Noncovalent PEGylation through protein-polyelectrolyte interaction: kinetic experiment and molecular dynamics simulation. J. Phys. Chem. B. 121 (28), 6785–6791. 10.1021/acs.jpcb.7b02741 28650657

[B88] Kusterle M. Jevševar S. Porekar V. G. (2008). Size of pegylated protein conjugates studied by various methods. Acta Chim. Slov. 55 (3), 594–601.

[B89] Lainka E. Hershfield M. S. Santisteban I. Bali P. Seibt A. Neubert J. (2005). Polyethylene glycol-conjugated adenosine deaminase (ADA) therapy provides temporary immune reconstitution to a child with delayed-onset ADA deficiency. Clin. Vaccine Immunol. 12 (7), 861–866. 10.1128/CDLI.12.7.861-866.2005 PMC1182205 16002636

[B90] Lakshmanan A. Farhadi A. Nety S. P. Lee-Gosselin A. Bourdeau R. W. Maresca D. (2016). Molecular engineering of acoustic protein nanostructures. ACS Nano. 10 (8), 7314–7322. 10.1021/acsnano.6b03364 27351374 PMC6058967

[B91] Lawrence P. B. Price J. L. (2016). How PEGylation influences protein conformational stability. Curr. Opin. Chem. Biol. 34, 88–94. 10.1016/j.cbpa.2016.08.006 27580482 PMC5107330

[B92] Lee H. Ho Jang I. Ho Ryu S. Park T. G. (2003). N-terminal site-specific mono-PEGylation of epidermal growth factor. Pharm. Res. 20 (818), 818–825. 10.1023/A:1023402123119 12751640

[B93] Lee J. I. Eisenberg S. P. Rosendahl M. S. Chlipala E. A. Brown J. D. Doherty D. H. (2013). Site-specific PEGylation enhances the pharmacokinetic properties and antitumor activity of interferon beta-1b. J. Interf. Cytokine Res. 33 (12), 769–777. 10.1089/jir.2012.0148 PMC3868373 23962003

[B94] Lee J. P. Kassianidou E. MacDonald J. I. Francis M. B. Kumar S. (2016). N-terminal specific conjugation of extracellular matrix proteins to 2-pyridinecarboxaldehyde functionalized polyacrylamide hydrogels. Biomaterials 102, 268–276 . 10.1016/j.biomaterials.2016.06.022 27348850 PMC4939314

[B95] Lee J. Oh E. T. Choi M. H. Kim H. G. Park H. J. Kim C. (2018). Dual-functional cyclic peptide switch on mesoporous nanocontainers for selective CD44 targeting and on-off gatekeeping triggered by conformational transformation. New J. Chem. 42, 12938–12944. 10.1039/c8nj02179a

[B96] Leung M. K. M. Hagemeyer C. E. Johnston A. P. R. Gonzales C. Kamphuis M. M. J. Ardipradja K. (2012). Bio-click chemistry: enzymatic functionalization of PEGylated capsules for targeting applications. Angew. Chemie Int. Ed. 51 (29), 7132–7136. 10.1002/anie.201203612 22744941

[B97] Li X. Zhang L. Hall S. E. Tam J. P. (2000). A new ligation method for N-terminal tryptophan-containing peptides using the Pictet-Spengler reaction. Tetrahedron Lett. 41 (2000), 4069–4073. 10.1016/S0040-4039(00)00592-X

[B98] Li X. Y. Li T. H. Guo J. S. Wei Y. Jing X. Chen X. S. (2012). PEGylation of bovine serum albumin using click chemistry for the application as drug carriers. Biotechnol. Prog. 28 (3), 856–861. 10.1002/btpr.1526 22275125

[B99] Lin S. He C. (2018). A mini-review on the enzyme-mediated manipulation of proteins/peptides. Chin. Chem. Lett. 28 (3), 856–861. 10.1016/j.cclet.2018.05.006

[B100] Liu C. C. Schultz P. G. (2010). Adding new chemistries to the genetic code. Annu. Rev. Biochem. 79, 413–444. 10.1146/annurev.biochem.052308.105824 20307192

[B101] Liu J. Chen Q. Rozovsky S. (2017). Utilizing selenocysteine for expressed protein ligation and bioconjugations. J. Am. Chem. Soc. 139 (9), 3430–3437 10.1021/jacs.6b1099 28186733 PMC5824972

[B102] Lou B. De Beuckelaer A. Dakwar G. R. Remaut K. Grooten J. Braeckmans K. (2018). Post-PEGylated and crosslinked polymeric ssRNA nanocomplexes as adjuvants targeting lymph nodes with increased cytolytic T cell inducing properties. J. Control. Release. 284, 73–83. 10.1016/j.jconrel.2018.06.010 29908893

[B103] Lyseng-Williamson K. A. (2011). Pegloticase: in treatment-refractory chronic gout. Drugs. 71 (16), 2179–2192. 10.2165/11202830-000000000-00000 22035516

[B104] Ma C. Zhang X. Wang K. Zhang X. Zhou Y. Liu H. (2015). A biocompatible cross-linked fluorescent polymer prepared via ring-opening PEGylation of 4-arm PEG-amine, itaconic anhydride, and an AIE monomer. Polym. Chem. 6, 3634–3640. 10.1039/c5py00111k

[B105] Macdonald J. I. Munch H. K. Moore T. Francis M. B. (2015). One-step site-specific modification of native proteins with 2-pyridinecarboxyaldehydes. Nat. Chem. Biol. 11 (5), 326–331. 10.1038/nchembio.1792 25822913

[B106] Macdougall I. C. Robson R. Opatrna S. Liogier X. Pannier A. Jordan P. (2006). Pharmacokinetics and pharmacodynamics of intravenous and subcutaneous continuous erythropoietin receptor activator (C.E.R.A.) in patients with chronic kidney disease. Clin. J. Am. Soc Nephrol. 1 (6), 1211–1215. 10.2215/CJN.00730306 17699350

[B107] Mariniello L. Porta R. (2005). Transglutaminases as biotechnological tools. Prog. Exp. Tumor Res. 38, 74–91. 10.1159/000084240 15746536

[B108] Marsac Y. Cramer J. Olschewski D. Alexandrov K. Becker C. F. W. (2006). Site-specific attachment of polyethylene glycol-like oligomers to proteins and peptides. Bioconjug. Chem. 17, 6, 1492–1498. 10.1021/bc0601931 17105228

[B109] Meneguetti G. P. Santos J. H. P. M. Obreque K. M. T. Vaz Barbosa C. M. Monteiro G. Farsky S. H. P. (2019). Novel site-specific PEGylated L-asparaginase. PloS One 14 (2), e0211951. 10.1371/journal.pone.0211951 30753228 PMC6372183

[B110] Mero A. Spolaore B. Veronese F. M. Fontana A. (2009). Transglutaminase-mediated PEGylation of proteins: direct identification of the sites of protein modification by mass spectrometry using a novel monodisperse PEG. Bioconjug. Chem. 20 (2), 384–389. 10.1021/bc800427n 19186937

[B111] Mero A. Ishino T. Chaiken I. Veronese F. M. Pasut G. (2011). Multivalent and flexible PEG-nitrilotriacetic acid derivatives for non-covalent protein pegylation. Pharm. Res. 28 (10), 2412–2421 10.1007/s11095-011-0468-8 21611874

[B112] Mero A. Grigoletto A. Maso K. Yoshioka H. Rosato A. Pasut G. (2016). Site-selective enzymatic chemistry for polymer conjugation to protein lysine residues: PEGylation of G-CSF at lysine-41. Polym. Chem. 7, 6545–6553 10.1039/c6py01616b

[B113] Milton Harris J. Chess R. B. (2003). Effect of pegylation on pharmaceuticals. Nat. Rev. Drug Discovery 2, 214–221. 10.1038/nrd1033 12612647

[B114] Milton Harris J. Martin N. E. Modi M. (2001). Pegylation: a novel process for modifying pharmacokinetics. Clin. Pharmacokinet. 10.2165/00003088-200140070-00005 11510630

[B115] Mirzaei H. Kazemi B. Bandehpour M. Shoari A. Asgary V. Ardestani M. S. (2016). Computational and nonglycosylated systems: a simpler approach for development of nanosized PEGylated proteins. Drug Des. Devel. Ther. 10, 1193–1200 10.2147/DDDT.S98323 PMC4801162 27042012

[B116] Mishra P. Nayak B. Dey R. K. (2016). PEGylation in anti-cancer therapy: An overview. Asian J. Pharm. Sci. (11) 3, 337–348 10.1016/j.ajps.2015.08.011

[B117] Mitchell S. F. Lorsch J. R. (2014). Protein derivitization-expressed protein ligation. Methods Enzymol. 536, 95–108 10.1016/B978-0-12-420070-8.00009-X 24423270

[B118] Mittal N. Sun D. X. Seidel D. (2014). Conjugate-base-stabilized brønsted acids: catalytic enantioselective pictet-spengler reactions with unmodified tryptamine. Org. Lett. 16 (3), 1012–1015 10.1021/ol403773a 24446703

[B119] Moelbert S. (2004). Correlation between sequence hydrophobicity and surface-exposure pattern of database proteins. Protein Sci. 13 (3), 752–762 10.1110/ps.03431704 14767075 PMC2286732

[B120] Molineux G. (2004). The design and development of pegfilgrastim (PEG-rmetHuG-CSF, Neulasta&#174);. Curr. Pharm. Des. 10 (11), 1235–1244 10.2174/1381612043452613 15078138

[B121] Mu Q. Hu T. Yu J. (2013). Molecular insight into the steric shielding effect of PEG on the conjugated staphylokinase: biochemical characterization and molecular dynamics simulation. PloS One 8 (7), e68559. 10.1371/journal.pone.0068559 23874671 PMC3715476

[B122] Mueller C. Capelle M. A. H. Arvinte T. Seyrek E. Borchard G. (2011a). Noncovalent pegylation by dansyl-poly(ethylene glycol)s as a new means against aggregation of salmon calcitonin. J. Pharm. Sci. 100 (5), 1648–1662 10.1002/jps.22401 21374604

[B123] Mueller C. Capelle M. A. H. Arvinte T. Seyrek E. Borchard G. (2011b). Tryptophan-mPEGs: Novel excipients that stabilize salmon calcitonin against aggregation by non-covalent PEGylation. Eur. J. Pharm. Biopharm. 79 (3), 646–645 10.1016/j.ejpb.2011.06.003 21703347

[B124] Mueller C. Capelle M. A. H. Seyrek E. Martel S. Carrupt P. A. Arvinte T. (2012). Noncovalent PEGylation: different effects of dansyl-, l-tryptophan-, phenylbutylamino-, benzyl- and cholesteryl-PEGs on the aggregation of salmon calcitonin and lysozyme. J. Pharm. Sci. 101 (6), 1995–2008. 10.1002/jps.23110 22447529

[B125] Muneeruddin K. Bobst C. E. Frenkel R. Houde D. Turyan I. Sosic Z. (2017). Characterization of a PEGylated protein therapeutic by ion exchange chromatography with on-line detection by native ESI MS and MS/MS. Analyst. 142 (2), 336–344. 10.1039/c6an02041k 27965993

[B126] Nguyen D. P. Mahesh M. Elsässer S. J. Hancock S. M. Uttamapinant C. Chin J. W. (2014a). Genetic encoding of photocaged cysteine allows photoactivation of TEV protease in live mammalian cells. J. Am. Chem. Soc. 136 (6), 2240–2243. 10.1021/ja412191m 24479649 PMC4333589

[B127] Nguyen G. K. T. Wang S. Qiu Y. Hemu X. Lian Y. Tam J. P. (2014b). Butelase 1 is an Asx-specific ligase enabling peptide macrocyclization and synthesis. Nat. Chem. Biol. 10 (9), 732–738. 10.1038/nchembio.1586 25038786

[B128] Nguyen G. K. T. Cao Y. Wang W. Liu C. F. Tam J. P. (2015). Site-Specific N-terminal labeling of peptides and proteins using Butelase 1 and thiodepsipeptide. Angew. Chemie Int. Ed. 54 (52), 15694–15698. 10.1002/anie.201506810 26563575

[B129] Nguyen G. K. T. Qiu Y. Cao Y. Hemu X. Liu C. F. Tam J. P. (2016). Butelase-mediated cyclization and ligation of peptides and proteins. Nat. Protoc. 11 (10), 1977–1988. 10.1038/nprot.2016.118 27658013

[B130] Obermeyer A. C. Olsen B. D. (2015). Synthesis and application of protein-containing block copolymers. ACS Macro Lett. 4, 1, 101–110. 10.1021/mz500732e 35596389

[B131] Obermeyer A. C. Jarman J. B. Francis M. B. (2014). N-terminal modification of proteins with o -aminophenols. J. Am. Chem. Soc. 136 (27), 9572–9579. 10.1021/ja500728c 24963951 PMC4353012

[B132] Oclon E. Solomon G. Hayouka Z. Salame T. M. Goffin V. Gertler A. (2018). Novel reagents for human prolactin research: Large-scale preparation and characterization of prolactin receptor extracellular domain, non-pegylated and pegylated prolactin and prolactin receptor antagonist. Protein Eng. Des. Sel. 31 (1), 7–16. 10.1093/protein/gzx062 29281090

[B133] Pelham R. W. Nix L. C. Chavira R. E. Cleveland M. V. Stetson P. (2008). Clinical trial: Single- and multiple-dose pharmacokinetics of polyethylene glycol (PEG-3350) in healthy young and elderly subjects. Aliment. Pharmacol. Ther. 28 (2), 256–265. 10.1111/j.1365-2036.2008.03727.x 18462266

[B134] Penczek S. Pretula J. B. (2016). “Ring-Opening Polymerization,” in Reference Module in Chemistry, Molecular Sciences and Chemical Engineering. Lodz, Poland: Center of Molecular and Macromolecular Studies of Polish Academy of Science

[B135] Plaks J. G. Falatach R. Kastantin M. Berberich J. A. Kaar J. L. (2015). Multisite clickable modification of proteins using lipoic acid ligase. Bioconjug. Chem. 26 (6), 1104–1112. 10.1021/acs.bioconjchem.5b00161 25982177

[B136] Polevoda B. Sherman F. (2003). N-terminal acetyltransferases and sequence requirements for N-terminal acetylation of eukaryotic proteins. J. Mol. Biol. 325 (4), 595–622. 10.1016/S0022-2836(02)01269-X 12507466

[B137] Popp M. W. L. Ploegh H. L. (2011). Making and breaking peptide bonds: Protein engineering using sortase. Angew. Chemie Int. Ed. 50 (22), 5024–5032. 10.1002/anie.201008267 21538739

[B138] Puthenveetil S. Liu D. S. White K. A. Thompson S. Ting A. Y. (2009). Yeast display evolution of a kinetically efficient 13-amino acid substrate for lipoic acid ligase. J. Am. Chem. Soc. 131 (45), 16430–16438. 10.1021/ja904596f 19863063 PMC2799336

[B139] Qi Y. Chilkoti A. (2015). Protein-polymer conjugation-moving beyond PEGylation. Curr. Opin. Chem. Biol. 28, 181–193. 10.1016/j.cbpa.2015.08.009 26356631 PMC4624571

[B140] Qin X. Li J. Li Y. Gan Y. Huang H. Liang C. J. (2017). Isoform separation and structural identification of mono-PEGylated recombinant human growth hormone (PEG-rhGH) with pH gradient chromatography. J. Chromatogr. B. Analyt. Technol. Biomed. Life Sci. 1044–1045: 206–213. 10.1016/j.jchromb.2016.12.014 28153672

[B141] Quémener D. Davis T. P. Barner-Kowollik C. Stenzel M. H. (2006). RAFT and click chemistry: a versatile approach to well-defined block copolymers. Chem. Commun. 5051–5053. 10.1039/b611224b 17146524

[B142] Rachel N. M. Pelletier J. N. (2013). Biotechnological applications of transglutaminases. Biomolecules. 3 (4), 870–888. 10.3390/biom3040870 24970194 PMC4030973

[B143] Raj M. Wu H. Blosser S. L. Vittoria M. A. Arora P. S. (2015). Aldehyde capture ligation for synthesis of native peptide bonds. J. Am. Chem. Soc. 137 (21), 6932–6940. 10.1021/jacs.5b03538 25966041

[B144] Reddington S. C. Howarth M. (2015). Secrets of a covalent interaction for biomaterials and biotechnology: SpyTag and SpyCatcher. Curr. Opin. Chem. Biol. 29, 94–99 10.1016/j.cbpa.2015.10.002 26517567

[B145] Reichert C. Borchard G. (2016). Noncovalent PEGylation, an innovative subchapter in the field of protein modification. J. Pharm. Sci. 105 (2), 386–390. 10.1002/jps.24692 26523632

[B146] Roberts M. J. Bentley M. D. Harris J. M. (2012). Chemistry for peptide and protein PEGylation. Adv. Drug Deliv. Rev. 54 (4), 459–476. 10.1016/j.addr.2012.09.025 12052709

[B147] Rosen C. B. Francis M. B. (2017). Targeting the N terminus for site-selective protein modification. Nat. Chem. Biol. 13 (7), 697–705. 10.1038/nchembio.2416 28632705

[B148] Roskos L. K. Lum P. Lockbaum P. Schwab G. Yang B. B. (2006). Pharmacokinetic/pharmacodynamic modeling of pegfilgrastim in healthy subjects. J. Clin. Pharmacol. 46 (7), 747–757. 10.1177/0091270006288731 16809800

[B149] Rostovtsev V. V. Green L. G. Fokin V. V. Sharpless K. B. (2002). A stepwise huisgen cycloaddition process: Copper(I)-catalyzed regioselective “ligation” of azides and terminal alkynes. Angew. Chemie Int. Ed. 41 (14), 2596–2599. 10.1002/1521-3773(20020715)41:14<2596::AID-ANIE2596>3.0.CO;2-4 12203546

[B150] Rouhani M. Khodabakhsh F. Norouzian D. Cohan R. A. Valizadeh V. (2018). Molecular dynamics simulation for rational protein engineering: Present and future prospectus. J. Mol. Graph. Model. 84, 43–53. 10.1016/j.jmgm.2018.06.009 29909273

[B151] Santos J. H. P. M. Torres-Obreque K. M. Meneguetti G. P. Amaro B. P. Rangel-Yagui C. O. (2018). Protein PEGylation for the design of biobetters: From reaction to purification processes. Braz. J. Pharm. Sci. 54, e01009. 10.1590/s2175-97902018000001009

[B152] Santos J. H. P. M. Carretero G. Ventura S. P. M. Converti A. Rangel-Yagui C. O. (2019). PEGylation as an efficient tool to enhance cytochrome c thermostability: a kinetic and thermodynamic study. J. Mater. Chem. B. 7, 4432–4439 10.1039/c9tb00590k

[B153] Sasaki T. Kodama K. Suzuki H. Fukuzawa S. Tachibana K. (2008). N-terminal labeling of proteins by the Pictet-Spengler reaction. Bioorg. Med. Chem. Lett. 18 (16), 4550–4553. 10.1016/j.bmcl.2008.07.033 18667304

[B154] Sathyamoorthy N. Magharla D. D. (2017). Clinical implications of molecular PEGylation on therapeutic proteins. J. Basic Clin. Pharm. 8, 87–90.

[B155] Sato H. (2002). Enzymatic procedure for site-specific pegylation of proteins. Adv. Drug Deliv. Rev. 54 (4), 487–504. 10.1016/S0169-409X(02)00024-8 12052711

[B156] Scaramuzza S. Tonon G. Olianas A. Messana I. Schrepfer R. Orsini G. (2012). A new site-specific monoPEGylated filgrastim derivative prepared by enzymatic conjugation: production and physicochemical characterization. J. Controlled Release. 164 (3), 355–363. 10.1016/j.jconrel.2012.06.026 22735238

[B157] Schiavon O. Caliceti P. Ferruti P. Veronese F. M. (2000). Therapeutic proteins: a comparison of chemical and biological properties of uricase conjugated to linear or branched poly(ethylene glycol) and poly(N- acryloylmorpholine). Farmaco. 55 (4), 264–269. 10.1016/S0014-827X(00)00031-8 10966157

[B158] Schmidt M. Toplak A. Quaedflieg P. J. Nuijens T. (2017). Enzyme-mediated ligation technologies for peptides and proteins. Curr. Opin. Chem. Biol. 38, 1–7. 10.1016/j.cbpa.2017.01.017 28229906

[B159] Schoene C. Fierer J. O. Bennett S. P. Howarth M. (2014). SpyTag/Spycatcher cyclization confers resilience to boiling on a mesophilic enzyme. Angew. Chemie Int. Ed. 53 (24), 6101–6114. 10.1002/anie.201402519 PMC4286826 24817566

[B160] Schoene C. Bennett S. P. Howarth M. (2016). SpyRing interrogation: analyzing how enzyme resilience can be achieved with phytase and distinct cyclization chemistries. Sci. Rep. 6, 21151. 10.1038/srep21151 26861173 PMC4748275

[B161] Schumacher D. Helma J. Mann F. A. Pichler G. Natale F. Krause E. (2015). Versatile and efficient site-specific protein functionalization by Tubulin tyrosine ligase. Angew. Chemie Int. Ed. 54 (46), 13787–13791. 10.1002/anie.201505456 26404067

[B162] Sharma M. Hire R. S. Hadapad A. B. Gupta G. D. Kumar V. (2017). PEGylation enhances mosquito-larvicidal activity of Lysinibacillus sphaericus binary toxin. Bioconjug. Chem. 28 (2), 410–418. 10.1021/acs.bioconjchem.6b00565 28118708

[B163] Shekhawat R. Shah C. K. Patel A. Srinivasan S. Kapoor P. Patel S. (2019). Structural similarity, characterization of Poly Ethylene Glycol linkage and identification of product related variants in biosimilar pegfilgrastim. PloS One 14 (3), e0212622. 10.1371/journal.pone.0212622 30865643 PMC6415886

[B164] Shi H. Shi Q. Oswald J. T. Gao Y. Li L. Li Y. (2018). Site-specific PEGylation of human growth hormone by mutated sortase A. Chem. Res. Chin. Univ. 34 (3), 428–433. 10.1007/s40242-018-8023-3

[B165] Soares A. L. Guimarães G. M. Polakiewicz B. Pitombo R. N. D. M. Abrahão-Neto J. (2002). Effects of polyethylene glycol attachment on physicochemical and biological stability of E. coli L-asparaginase. Int. J. Pharm. 237 (1–2), 163–170. 10.1016/S0378-5173(02)00046-7 11955814

[B166] Song I. T. Lee M. Lee H. Han J. Jang J. H. Lee M. S. (2016). PEGylation and HAylation via catechol: α-Amine-specific reaction at N-terminus of peptides and proteins. Acta Biomater. 43, 50–60. 10.1016/j.actbio.2016.07.018 27424082

[B167] Sosic A. Pasqualin M. Pasut G. Gatto B. (2014). Enzymatic formation of PEGylated oligonucleotides. Bioconjug. Chem. 25 (2), 433–441. 10.1021/bc400569z 24450424

[B168] Spears B. R. Waksal J. McQuade C. Lanier L. Harth E. (2013). Controlled branching of polyglycidol and formation of protein-glycidol bioconjugates via a graft-from approach with “pEG-like” arms. Chem. Commun. 49 (24), 2394–2396. 10.1039/c3cc38369e 23370543

[B169] Spolaore B. Raboni S. Satwekar A. A. Grigoletto A. Mero A. Montagner I. M. (2016). Site-specific transglutaminase-mediated conjugation of interferon α-2b at glutamine or lysine residues. Bioconjug. Chem. 27 (11), 2695–2706. 10.1021/acs.bioconjchem.6b00468 27731976

[B170] Stidl R. Fuchs S. Bossard M. Siekmann J. Turecek P. L. Putz M. (2016). Safety of PEGylated recombinant human full-length coagulation factor VIII (BAX 855) in the overall context of PEG and PEG conjugates. Haemophilia. 22 (1), 54–64. 10.1111/hae.12762 PMC4737295 26219204

[B171] Strop P. (2014). Versatility of microbial transglutaminase. Bioconjug. Chem. 25(5), 855–62. 10.1021/bc500099v 24694238

[B172] Su Y. C. Chen B. M. Chuang K. H. Cheng T. L. Roffler S. R. (2010). Sensitive quantification of PEGylated compounds by second-generation anti-poly(ethylene glycol) monoclonal antibodies. Bioconjug. Chem. 21 (7), 1264–1270. 10.1021/bc100067t 20536171

[B173] Taki M. Sisido M. (2007). Leucyl/phenylalanyl(L/F)-tRNA-protein transferase-mediated aminoacyl transfer of a nonnatural amino acid to the N-terminus of peptides and proteins and subsequent functionalization by bioorthogonal reactions. Biopolymers Pept. Sci. Section. 88 (2), 263–271. 10.1002/bip.20678 17216634

[B174] Thordarson P. Le Droumaguet B. Velonia K. (2006). Well-defined protein-polymer conjugates - synthesis and potential applications. Appl. Microbiol. Biotechnol. 73 (2), 243–54. 10.1007/s00253-006-0574-4 17061132

[B175] Tian J. Sun J. Li Z. (2018). Biomimetic pegylated polypeptoids with thermoresponsive properties. Polymer (Guildf). 138, 132–138. 10.1016/j.polymer.2018.01.034

[B176] Tilley S. D. Francis M. B. (2006). Tyrosine-selective protein alkylation using ??-allylpalladium complexes. J. Am. Chem. Soc. 128 (4), 1080–1081. 10.1021/ja057106k 16433516

[B177] Tomita U. Yamaguchi S. Maeda Y. Chujo K. Minamihata K. Nagamune T. (2013). Protein cell-surface display through in situ enzymatic modification of proteins with a poly(Ethylene glycol)-lipid. Biotechnol. Bioeng. 110 (10), 2785–2789. 10.1002/bit.24933 23592269

[B178] Tsukiji S. Nagamune T. (2009). Sortase-mediated ligation: A gift from gram-positive bacteria to protein engineering. ChemBioChem. 10 (5), 787–798. 10.1002/cbic.200800724 19199328

[B179] Tucker B. S. Coughlin M. L. Figg C. A. Sumerlin B. S. (2017). Grafting-from proteins using metal-free PET-RAFT polymerizations under mild visible-light irradiation. ACS Macro Lett. 6, 4, 452–457 10.1021/acsmacrolett.7b00140 35610863

[B180] Turecek P. L. Bossard M. J. Schoetens F. Ivens I. A. (2016). PEGylation of biopharmaceuticals: a review of chemistry and nonclinical safety information of approved drugs. J. Pharm. Sci. 105 (2), 460–475. 10.1016/j.xphs.2015.11.015 26869412

[B181] Uprety R. Luo J. Liu J. Naro Y. Samanta S. Deiters A. (2014). Genetic encoding of caged cysteine and caged homocysteine in bacterial and mammalian cells. ChemBioChem. 15 (12), 1793–1799. 10.1002/cbic.201400073 24976145

[B182] van Leeuwen L. A. G. Hinchy E. C. Murphy M. P. Robb E. L. Cochemé H. M. (2017). Click-PEGylation – A mobility shift approach to assess the redox state of cysteines in candidate proteins. Free Radic. Biol. Med. 108, 374–382. 10.1016/j.freeradbiomed.2017.03.037 28366801 PMC5488967

[B183] Vernet E. Popa G. Pozdnyakova I. Rasmussen J. E. Grohganz H. Giehm L. (2016). Large-scale biophysical evaluation of protein PEGylation effects: in vitro properties of 61 protein entities. Mol. Pharm. 13 (5), 1587–1598. 10.1021/acs.molpharmaceut.6b00049 27043713

[B184] Villar H. O. Koehler R. T. (2000). Amino acid preferences of small, naturally occurring polypeptides. Biopolymers. 53 (3), 226–232. 10.1002/(SICI)1097-0282(200003)533 < 226AID-BIP2 > 3.0.CO;2-# 10679627 10.1002/(SICI)1097-0282(200003)53:3<226::AID-BIP2>3.0.CO;2-#

[B185] Wallat J. D. Rose K. A. Pokorski J. K. (2014). Proteins as substrates for controlled radical polymerization. Polym. Chem. 5, 1545–1558. 10.1039/c3py01193c

[B186] Wan Q. Wang K. Du H. Huang H. Liu M. Deng F. (2015). A rather facile strategy for the fabrication of PEGylated AIE nanoprobes. Polym. Chem. 6, 5288–5294 10.1039/c5py00735f

[B187] Wan X. Zhang J. Yu W. Shen L. Ji S. Hu T. (2017). Effect of protein immunogenicity and PEG size and branching on the anti-PEG immune response to PEGylated proteins. Process. Biochem. 10.1016/j.procbio.2016.09.029

[B188] Wang Y. Youngster S. Grace M. Bausch J. Bordens R. Wyss D. F. (2002). Structural and biological characterization of pegylated recombinant interferon alpha-2b and its therapeutic implications. Adv. Drug Deliv. Rev. 54 (4), 547–570. 10.1016/S0169-409X(02)00027-3 12052714

[B189] Wang S. J. Wu S. T. Gokemeijer J. Fura A. Krishna M. Morin P. (2012). Attribution of the discrepancy between ELISA and LC-MS/MS assay results of a PEGylated scaffold protein in post-dose monkey plasma samples due to the presence of anti-drug antibodies. Anal. Bioanal. Chem. 402 (3), 1229–1239. 10.1007/s00216-011-5527-9 22130720

[B190] Wang J. Wang Y. Wang X. Zhang D. Wu S. Zhang G. (2016). Enhanced thermal stability of lichenase from Bacillus subtilis 168 by SpyTag/SpyCatcher-mediated spontaneous cyclization. Biotechnol. Biofuels. 9, 79. 10.1186/s13068-016-0490-5 27034717 PMC4815112

[B191] Wang H. H. Altun B. Nwe K. Tsourkas A. (2017). Proximity-based sortase-mediated ligation. Angew. Chemie Int. Ed. 56 (19), 5349–5352. 10.1002/anie.201701419 PMC5537000 28374553

[B192] Wiegandt A. Meyer B. (2014). Unambiguous characterization of N-glycans of monoclonal antibody cetuximab by integration of LC-MS/MS and 1H NMR spectroscopy. Anal. Chem. 86 (10), 4807–4814. 10.1021/ac404043g 24725217

[B193] Wiita A. P. Hsu G. W. Lu C. M. Esensten J. H. Wells J. A. (2014). Circulating proteolytic signatures of chemotherapy-induced cell death in humans discovered by N-terminal labeling. Proc. Natl. Acad. Sci. 111 (21), 7594–7599. 10.1073/pnas.1405987111 24821784 PMC4040605

[B194] Wissner R. F. Batjargal S. Fadzen C. M. Petersson E. J. (2013). Labeling proteins with fluorophore/thioamide Förster resonant energy transfer pairs by combining unnatural amino acid mutagenesis and native chemical ligation. J. Am. Chem. Soc. 135 (17), 6529–6540. 10.1021/ja4005943 23594264 PMC3721677

[B195] Wright M. H. Heal W. P. Mann D. J. Tate E. W. (2010). Protein myristoylation in health and disease. J. Chem. Biol. 3 (1), 19–35. 10.1007/s12154-009-0032-8 19898886 PMC2816741

[B196] Wu L. Chen J. Wu Y. Zhang B. Cai X. Zhang Z. (2017). Precise and combinatorial PEGylation generates a low-immunogenic and stable form of human growth hormone. J. Control. Release. 249, 84–93. 10.1016/j.jconrel.2017.01.029 28131652

[B197] Xiao S. J. Wieland M. Brunner S. (2005). Surface reactions of 4-aminothiophenol with heterobifunctional crosslinkers bearing both succinimidyl ester and maleimide for biomolecular immobilization. J. Colloid Interface Sci. 290 (1), 172–183. 10.1016/j.jcis.2005.04.014 15925374

[B198] Xiao Y. P. Zhang J. Liu Y. H. Chen X. C. Yu Q. Y. Luan C. R. (2018). Ring-opening polymerization of diepoxides as an alternative method to overcome PEG dilemma in gene delivery. Polymer (Guildf). 134, 53–62. 10.1016/j.polymer.2017.11.059

[B199] Xiaojiao S. Corbett B. Macdonald B. Mhaskar P. Ghosh R. (2016). Modeling and optimization of protein pegylation. Ind. Eng. Chem. Res. 55, 45, 11785–11794. 10.1021/acs.iecr.6b03122

[B200] Xu L. Q. Li N. N. Zhang B. Chen J. C. Kang E. T. (2015). Pegylated fluorescent nanoparticles from one-pot atom transfer radical polymerization and “click chemistry”. Polymers (Basel). 7 (10), 2119–2130. 10.3390/polym7101504

[B201] Xu D. Smolin N. Shaw R. K. Battey S. R. Tao A. Huang Y. (2018a). Molecular insights into the improved clinical performance of PEGylated interferon therapeutics: a molecular dynamics perspective. RSC Adv. 8, 2315–2322. 10.1039/c7ra12480e PMC9077387 35541455

[B202] Xu D. Zeng S. Liu M. Chen J. Huang H. Deng F. (2018b). Preparation of PEGylated and biodegradable fluorescent organic nanoparticles with aggregation-induced emission characteristics through direct ring-opening polymerization. J. Taiwan Inst. Chem. Eng. 95, 234–240. 10.1016/j.jtice.2018.07.008

[B203] Yang T. Cui F. Choi M. K. Cho J. W. Chung S. J. Shim C. K. (2007). Enhanced solubility and stability of PEGylated liposomal paclitaxel: in vitro and in vivo evaluation. Int. J. Pharm. 338 (1–2), 317–326. 10.1016/j.ijpharm.2007.02.011 17368984

[B204] Yin L. Su C. Ren T. Meng X. Shi M. Paul Fawcett J. (2017). MSAll strategy for comprehensive quantitative analysis of PEGylated-doxorubicin, PEG and doxorubicin by LC-high resolution q-q-TOF mass spectrometry coupled with all window acquisition of all fragment ion spectra. Analyst. 142 (22), 4279–4288. 10.1039/c7an00470b 29022970

[B205] Zaghmi A. Greschner A. A. Mendez-Villuendas E. Liu J. Y. de Haan H. W. Gauthier M. A. (2019). Determination of the degree of PEGylation of protein bioconjugates using data from proton nuclear magnetic resonance spectroscopy. Data Br. 25, 104037. 10.1016/j.dib.2019.104037 PMC6565728 31223640

[B206] Zakeri B. Fierer J. O. Celik E. Chittock E. C. Schwarz-Linek U. Moy V. T. (2012). Peptide tag forming a rapid covalent bond to a protein, through engineering a bacterial adhesin. Proc. Natl. Acad. Sci. 109 (12), E690–E697. 10.1073/pnas.1115485109 22366317 PMC3311370

[B207] Zhang W. Jiang Z. Wang L. Li C. Xia J. (2015). An open-label, randomized, multicenter dose-finding study of once-per-cycle pegfilgrastim versus daily filgrastim in Chinese breast cancer patients receiving TAC chemotherapy. Med. Oncol. 32 (5), 147. 10.1007/s12032-015-0537-7 25820754

[B208] Zhang Y. Park K.-Y. Suazo K. F. Distefano M. D. (2018). Recent progress in enzymatic protein labelling techniques and their applications. Chem. Soc. Rev. 47 (24), 9106–9136. 10.1039/C8CS00537K 30259933 PMC6289631

[B209] Zhao T. Cheng Y.-N. Tan H.-N. Liu J.-F. Xu H.-L. Pang G.-L. (2012). Site-specific chemical modification of human serum albumin with polyethylene glycol prolongs half-life and improves intravascular retention in mice. Biol. Pharm. Bull. 35 (3), 280–288. 10.1248/bpb.35.280 22382312

[B210] Zheng J. C. Lei N. He Q. C. Hu W. Jin J. G. Meng Y. (2012). PEGylation is effective in reducing immunogenicity, immunotoxicity, and hepatotoxicity of α-momorcharin *in vivo* . Immunopharmacol. Immunotoxicol. 34 (5), 866–873. 10.3109/08923973.2012.666979 22439816

